# Differences in inflammation and acute phase response but similar genotoxicity in mice following pulmonary exposure to graphene oxide and reduced graphene oxide

**DOI:** 10.1371/journal.pone.0178355

**Published:** 2017-06-01

**Authors:** Stefan Bengtson, Kristina B. Knudsen, Zdenka O. Kyjovska, Trine Berthing, Vidar Skaug, Marcus Levin, Ismo K. Koponen, Abhay Shivayogimath, Timothy J. Booth, Beatriz Alonso, Amaia Pesquera, Amaia Zurutuza, Birthe L. Thomsen, Jesper T. Troelsen, Nicklas R. Jacobsen, Ulla Vogel

**Affiliations:** 1 National Research Centre for the Working Environment, Copenhagen Ø, Denmark; 2 Department of Science and Environment, Roskilde University, Roskilde, Denmark; 3 National Institute of Occupational Health, Oslo, Norway; 4 Department of Micro- and Nanotechnology, Technical University of Denmark, Kgs. Lyngby, Denmark; 5 R&D Department, Graphenea S.A., San Sebastian, Spain; Centre National de la Recherche Scientifique, FRANCE

## Abstract

We investigated toxicity of 2–3 layered >1 μm sized graphene oxide (GO) and reduced graphene oxide (rGO) in mice following single intratracheal exposure with respect to pulmonary inflammation, acute phase response (biomarker for risk of cardiovascular disease) and genotoxicity. In addition, we assessed exposure levels of particulate matter emitted during production of graphene in a clean room and in a normal industrial environment using chemical vapour deposition. Toxicity was evaluated at day 1, 3, 28 and 90 days (18, 54 and 162 μg/mouse), except for GO exposed mice at day 28 and 90 where only the lowest dose was evaluated. GO induced a strong acute inflammatory response together with a pulmonary (*Serum-Amyloid A*, *Saa3)* and hepatic (*Saa1)* acute phase response. rGO induced less acute, but a constant and prolonged inflammation up to day 90. Lung histopathology showed particle agglomerates at day 90 without signs of fibrosis. In addition, DNA damage in BAL cells was observed across time points and doses for both GO and rGO. In conclusion, pulmonary exposure to GO and rGO induced inflammation, acute phase response and genotoxicity but no fibrosis.

## Background

Increasing interest in graphene-based applications is reflected in the global growth of graphene production and in the number of graphene patent publications through the last ten years [1,2]. Graphene-based nanoparticles are a source of concern regarding potential health hazards, similar to e.g. carbon nanotubes (CNT), despite the quite different 2-dimensional structure and large lateral size [3]. Inhaled graphene can penetrate the upper respiratory tract and deposits in the alveolar region [4,5]. Previous studies have shown that graphene particles can persist in the lung for up to 6 weeks and in some cases cause frustrated phagocytosis and long-term inflammation [5–8].

Inhalation of particles by e.g. air pollution is associated with risk of cardiovascular disease [9], where initiation of systemic acute phase response (APR) and increase in APR proteins in the blood (e.g. C-reactive protein and SAA) have been proposed as a pathogenic mechanisms [10,11]. Increased blood APR proteins are well-known risk factors for cardiovascular disease [12–14]. In mice, the acute phase gene Serum Amyloid A3 (*Saa3*) is expressed in lung and much less in hepatic tissue. Conversely, *Saa1* and *Saa2* are the main Saa isoforms expressed in the liver [15], although increased expression of Saa1 and Saa2 are also strongly increased in lungs following exposure to titanium dioxide, carbon black Printex90 (P90) and MWCNT [16–19]. These nanomaterials was shown to induce pulmonary APR with Saa3 being the most differentially regulated gene [10,11]. Therefore, we have used *Saa3* expression as a marker of pulmonary acute phase response [10,18;20–23]. Interestingly, only CNT exposure induced increased expression of both pulmonary (*Saa1*, *Saa2*, *Saa3*) and hepatic (*Saa1* and *Saa2*) APR genes [23,24]. We have shown that pulmonary inflammation, as a consequence of exposure to these carbon-based materials, is a predictor of pulmonary *Saa3* expression in mice [10]. Furthermore, we have provided evidence that the total surface area of these instilled particles correlates with the pulmonary *Saa3* expression [11,25]. Due to the sheet-like morphology and high surface area of graphene-based materials [3,26,27], it is relevant to assess the level of APR to estimate the risk of cardiovascular disease.

We have previously conducted genotoxicity assessment of related carbon-materials in both *in vitro* and *in vivo* models, where P90 and diesel exhaust particles (DEP) have been shown to induce genotoxicity *in vitro* and *in vivo* [16,21,22,28–32]. On the other hand, only limited genotoxicity of CNT was observed *in vitro* [29] while Poulsen et al. recently reported that the diameter of MWCNT was a predictor of genotoxicity *in vivo* [25].

Graphene materials comprise a group of materials including e.g. single or few-layered graphene, graphene oxide (GO) and reduced graphene oxide (rGO) [33,34], that are commonly produced [2] and widely used in *in vivo* toxicity testing [5,6,35–37]. We recently published an extensive physicochemical characterization of commercially available rGO and GO and evaluated the cytotoxicity and genotoxicity in the murine lung epithelial cell line FE1 [26]. GO (1–2 μm) and rGO (2–3 μm) were comparable in layer numbers (2–3 layers) and free of impurities (<1.5 wt %). GO generated high levels of reactive oxygen species (ROS), but neither GO nor rGO induced cytotoxicity or genotoxicity *in vitro*.

Occupational exposure to nanomaterials during manufacturing and handling has been reported previously [38,39]. Due to the properties of graphene in potential new electronic applications, techniques for large-area graphene production by e.g. chemical vapour deposition are becoming more relevant [40,41]. This method allows graphene manufacturing in a gas tight vacuum chamber thus minimizing dust exposure [42], however the chamber must be vented and opened in order to retrieve grown material. To assess the exposure to airborne particulate matter during manufacturing of graphene by chemical vapour deposition, we conducted exposure measurement at two different production sites employing this method.

In order to provide data for hazard assessment of occupational exposure to graphene-based materials, we assessed short and long-term toxicity of GO and rGO following single intratracheal exposure in mice. Work place exposure was assessed during the production of graphene using chemical vapour deposition at a small scale production site in clean room and at large scale production site in an industrial environment.

## Materials and methods

### Materials

Tested materials in this study included two commercial available graphene derivatives, one graphene oxide (GO) and one reduced graphene oxide (rGO) manufactured and supplied by Graphenea (San Sebastian, Spain). GO was synthesized by chemical exfoliation of synthetic graphite using a modified Hummer’s method and later chemically reduced with ascorbate. GO was delivered in a water suspension whereas rGO was in powder form (washed with methanol, filtered and air-dried under vacuum). A detailed description and an in-depth characterization of GO and rGO has been published previously [26].

Carbon Black Printex90 (P90), provided by Degussa (Frankfurt, Germany) was included as reference material (162μg/mouse) based on findings from previous studies showing inflammatory and genotoxic response in mice following single instillation [16]. We routinely include P90 as a reference material [21–23,43–47] andP90 has been described and characterized in detail [44,48].

### Material dispersions

GO, rGO and P90 were prepared in 0.2 μm filtered, γ-irradiated Nanopure Diamond UV water (Pyrogens: < 0,001 EU/ml, total organic carbon: < 3.0 ppb) added 0.1%Tween80^®^ (TW80) to a final concentration of particles of 3.24mg/ml. To achieve a homogenous dispersion, the final solution was then prepared by probe sonication on ice for 16 min with 10% amplitude (Branson Sonifier S-450D, Branson Ultrasonics Corp., Danbury, CT, USA) equipped with disruptor horn (model number 101–147–037). Following sonication, solution was further diluted to 1.08mg/ml and sonicated for 2 minutes. Dilution was further diluted to 0.36 mg/ml and sonicated 2 minutes. As vehicle control (VC), Nanopure water added 0.1% TW80 was prepared by procedure as described above. Suspensions were instilled in mice within 20 minutes after sonication.

### Hydrodynamic size, zeta potential and pH level

Immediately after sonication, 700 μl was transferred to a 4.5 ml polystyrene spectrophotometer cuvette. The hydrodynamic size of GO and rGO in vehicle was determined by dynamic light scattering (DLS) at concentrations: 3.24, 1.08 and 0.36 mg/ml, respectively. The hydrodynamic size (Z-average), size distribution (light intensity and number weighted distribution) and polydispersity index (PDI) were measured 6 times and mean was calculated. Viscosity was set to 0.97 mpa.s. (corresponding to 0.1% TW80). Refractive (R_i_) and absorption indices (R_a_) were used when transforming from light intensity distribution to number distribution for GO (R_i_: 1.7 R_a_: 2.0), rGO (R_i_: 2.8, R_a_: 2.0) and P90 (R_i_: 2.02, R_a_: 2.0). Zeta-potential and pH was determined by preparing GO, rGO and P90 in 0.1%TW80 (3.24 mg/ml) and sonicated as described above. A detailed description of hydrodynamic size, zeta-potential and pH measurements have been described previously [26].

### Animals

A total of 306 C57BL/6J female mice, 7-weeks old, delivered by Taconic Europe (Ejby, Denmark), were used in a main (pulmonary inflammation, acute phase response, genotoxicity and histopathology) and a second *in vivo* experiment (effect of pH on inflammation). All mice were randomly grouped according to particle exposure, dose and day of euthanasia. They were housed in polypropylene cages with sawdust bedding and enrichment. All mice had access to food (Altromin 1324) and tap water ad libitum. Temperature and humidity was controlled at 21 ± 1°C and 50 ± 10% respectively with a 12-h light and 12-h dark cycle. Daily observations of clinical signs of stress and discomfort were performed.

The mean bodyweight of mice at 8-weeks of age was 19.7 ± 1 g. Bodyweight was further monitored at 3–5 time points (*n* > 6) throughout the study period (S1 Table).

The animal experiments comply with the ARRIVE guidelines [49]. All procedures followed the guidelines for care and handling of laboratory animals according to the EC Directive 86/609/EEC, the Danish law and were approved by the Danish Animal Experiment Inspectorate (under the Danish Ministry of Justice), permission 2012/15-2934-00223561-1123.

### Study design

Mice were exposed to either VC, GO, rGO or the reference particle P90 by single intratracheal instillation (50 μl/mouse) under isoflurane sedation between 9 to 11 a.m, as previously described [50]. In the main experiment, mice (282 in total) were grouped based on exposure and dose level (VC groups *n* = 8, exposed groups *n* = 7). Mice used for histology were grouped with fewer mice (VC *n* = 3, exposed groups *n* = 6). The applied doses in main study were 18 μg, 54 μg and 162 μg/mouse. Mice were euthanized at 1, 3, 28 or 90 days post exposure.

In the second experiment, 24 mice were grouped in same study design based on exposure and dose level (VC groups and exposed groups, *n* = 6). Mice were exposed by intratracheal instillation (50 μl/mouse) to PBS (VC_PBS_), 0.1% TW80 (VC_TW80_), 18 μg/mouse of GO dispersed in PBS (GO_PBS_) or 18 μg/mouse of GO dispersed in 0.1% TW80 (GO_TW80_). Mice were euthanized 3 days post exposure.

### Necropsy and preparation of BAL samples

All mice were anesthetized by i.p. injection of 0.1 ml ZRF solution (Zoletil 250 mg, Rompun 20 mg/ml, Fentanyl 50mg/ml in sterile isotone saline). Blood was withdrawn from the heart and stabilized using 36 μl K_2_EDTA and followed by collection of BAL where lungs were flushed twice with 0.8 ml 0.9% sterile saline. Total BAL recovery was about 1.4 ml. BAL samples were immediately stored on ice until further preparation. BAL cells were prepared on glass slides and stained with May-Grünewald-Giemsa staining. Details about preparation method have been described previously [22]. Images of BAL cells were acquired at 100x on an Olympus BX 43 microscope with a Qimaging Retiga4000R camera. Uneven illumination in brightfield images was corrected using ImageJ [51] and the Calculator Plus plugin via the formula: Corrected image = (Image / background) * 255. The background image was a maximum projection of 3 background brightfield images without BAL cells.

### Cell composition in BAL fluid

Pulmonary inflammation was evaluated by analysis of cell composition of 200 cells in BAL fluid. Scoring was performed on blinded samples using light microscopy (Leitz Laborlux K) at 100x magnification using immersion oil. Counted cells were expressed as % observations based on distribution of the 200 cells multiplied with the total number of cells in each sample.

### RNA extraction

Total RNA was isolated from the left lung and lateral lobe of liver (6–23 mg). Total RNA was isolated from tissues by using Maxwell^®^ 16 LEV simplyRNA Tissue Kit (Promega Biotech AB, Sweden) according to the manufacturer’s instructions. The final RNA concentration for each sample was measured on Nanodrop 2000c (ThermoFischer Scientific, Denmark). Nucleic acid purity (A260/A280) was measured to 2.10 ± 0.007. Isolated RNA was stored at -80°C until further analysis.

### mRNA expression

Gene expression of *Saa3* mRNA levels in lungs and *Saa1* in liver was assessed for time points 1, 3, 28 and 90 days. cDNA synthesis was prepared from isolated RNA using Taq-man Reverse Transcriptation Reagent Kit (ThermoFischer Scientific, Denmark) according to manufacturer’s protocol. The relative gene expression of target genes *Saa1* and *Saa3* was by RT-qPCR on ViiA^™^ 7 (ThermoFischer Scientific, Denmark) and calculated by comparative method 2^-ΔCT^ [52]. The reference gene 18S was used for normalization. The nucleotide sequence of *Saa3* primers and probe were forward: 5’ GCC TGG GCT GCT AAA GTC AT 3’, reverse: 5’ TGC TCC ATG TCC CGT GAA C 3’ and probe: 50 FAM- TCT GAA CAG CCT CTC TGG CAT CGC T-TAMRA 3’ and *Saa1* (Mm00656927_g1). Target and reference genes were run in triplicate in 384-well reaction plates (ThermoFischer Scientific, Denmark) including–RT controls and negative controls without synthesized cDNA.

### SAA3 protein in in blood

The level of SAA3 protein in blood of mice was evaluated at day 3 for all doses by ELISA using Mouse SAA-3 ELISA (EZMSAA3-12K, Merck Millipore, Denmark) and conducted according to the manufacturer’s protocol. Plasma samples were pooled two at a time randomly to a final *n* = 3 (representing 6 mice in total). Absorbance (450 nm and 590 nm) was measured on Epoch^™^ microplate spectrophotometer (BioTek, Winooski, USA) within 5 minutes.

### Histopathology

Lung inflammation was also evaluated using histopathological analysis of lung tissue from mice at day 3 for VC and GO only (54 and 162 μg/mouse) and day 90 for VC, GO (18 μg/mouse) and rGO (18, 54 and 162 μg/mouse). Lungs were fixed by very slowly filling the trachea with formaldehyde (4%) under 30 cm water column pressure. Lungs were then stored in 4% formaldehyde for a minimum of 24 hours, before trimming and embedding in paraffin. The left lung lobe was divided in two halves and embedded in paraffin block, while all lobes from the right side were embedded in another block. After paraffin embedment, thin paraffin sections of 3 μm were cut and mounted on a glass slide and stained with hematoxylin and eosin using standard histological protocol followed by light microscopy examination. Images were acquired (10x and 40x) and corrected using protocol described for BAL cells.

### Genotoxicity

Genotoxicity was assessed in BAL, lung and liver from mice exposed to GO, rGO and P90 at all time points and for all available dose levels. DNA strand breaks were used as a marker for genotoxicity and were analyzed using comet assay on 20-well Trevigen Cometslides^™^ and automated scoring (Pathfinder^™^, IMSTAR, France). Samples related to each time point were placed on the same electrophoresis. Day-to-day variation and efficacy of each individual electrophoresis was validated by including A549 cells exposed to either PBS or H_2_O_2_ (60 μM) included on each slides (S3 Fig). A detailed description of sample preparation and further analysis has been described previously [53].

### Workplace exposure assessment

The potential exposure levels of graphene and other particulate matter emitted during specific work processes, at two production sites, during 8 hour working days were assessed. At both sites, graphene was produced by chemical vapour deposition using a commercial system Black Magic Enhanced CVD (BM Pro, AIXTRON, UK) allowing graphene growth on substrates of up to 4 inch. In the first case, measurements were performed during production of graphene situated in a clean room (~40 m^3^), class 10–100, ISO 9001-certified) with no local ventilation. We applied a second tier approach in the measurements [54]. The assessment was based on total number concentration of emitted airborne particles and further particle characterization by TEM imaging to verify origin of emissions. This approach was done in both Near Field (close to process, NF) and Far Field (background, FF) [55]. Particle number concentrations were measured using a Condensation Particle Counter (CPC3007, TSI Inc., MN, USA) and a DiSCmini (Testo SE & Co. KGaA, Germany). Samples for characterization were collected on holey-carbon coated copper grids using a Miniature Particle Sampler (MPS, Ecomesure, France) and analyzed using TEM (Titan E-Cell 80-300S ST TEM, FEI, NE, USA). The same approach was used in the second case where measurements were performed at a graphene production site in an industrial setting (room size ~500 m^3^) with no local ventilation. S1 Fig. shows a schematic overview of the room layout for both case measurements along with sampling positions.

### Statistics

Statistical analysis was performed in Minitab v.17.1.0 (Minitab Inc., State College, PA, USA), except for neutrophils where SAS^®^, version 9.3 for the Windows platform was used. All data are presented as mean ± standard error of the mean (Mean±SEM).

All statistical analyses were performed on log-transformed data, except for data on genotoxicity. The analyses of GO and rGO were performed for each day separately using two-way Analysis of Variance (ANOVA) with particle and dose level set as fixed category variables interacting with each other. In case of interaction, one-way ANOVA was performed including pairwise comparisons using Tukey’s adjusted p-values (significance level: 0.05). Analysis of data for P90 was performed using unpaired t-test at all time points. For cells in BAL, based a very high variation caused by numerous zero-values in the dataset for number of eosinophils and lymphocytes, statistical analysis was only performed on number of neutrophils, macrophages and total cells. For neutrophils, there were some samples with no neutrophils among the 200 cells counted. The total number of neutrophils in each of these samples was considered to be left censored at the total number of cells in the sample divided by 200. For neutrophils, the two-way ANOVA for each day with interaction between particle and dose were performed using the Lifereg procedure in SAS^®^. Statistical analysis of SAA3 in plasma was performed using Kruskal-Wallis multiple comparison with dose level set as fixed categorical variable for GO and rGO, respectively (significant level: 0.05, confidence interval: 0.95). Statistical analysis of DNA damage (%DNA) was performed on data normalized to the mean %DNA of PBS-exposed A549 cells on slides included in each electrophoresis (S3 Fig).

## Results

### Characterization of GO and rGO

A detailed physicochemical characterization of GO and rGO has been published previously [26]. An overview on key physicochemical characteristics is presented in Table 1. In brief, both GO and rGO mainly existed as 2–3 layered graphene. A clear tendency of wrinkling of rGO sheets and positions with additional 1–2 more layers were observed. The lateral size was determined to 2–3 μm and 1–2 μm for GO and rGO respectively. Surface area of rGO was 411 m^2^/g. As GO was delivered in a water suspension, BET surface area could not be determined. Both materials mainly consisted of C, O and H, where the C/O and C/H ratios were 1.4 and 1.7 for GO, 8.5 and 13.2 for rGO, respectively, indicating that GO had a high content of hydroxyl groups as expected. Inorganic impurities (< 1.5% in total) were mainly S, Mn and Si, with highest levels in rGO. GO was delivered in a water suspension and the level of organic impurities was negligible. Low levels of endotoxin were detected in GO (1.77 EU/mg) and rGO (1.05 EU/mg). This corresponded to a dose of 0.03–0.29 EU or 0.027–0.17 EU for GO and rGO, respectively in this present study. The levels were much lower than required for induction of pulmonary inflammation [56]. A lower hydrodynamic size for rGO compared to GO was observed (S4 Fig) that may be caused by higher degree of aggregation and sedimentation during DLS analysis.

**Table 1:** Characterization of the GO, rGO and the reference P90.

	GO	rGO	P90
**Number of layers**^a^	2–3^a^	2–3^a^	-
**Lateral size (μm)**^a^	2–3^a^	1–2^a^	0.009^b^
**Surface area (m**^**2**^**/g)**^a^	-	411^a^	338^c^
**C/O ratio**	1.4^a^	8.5^a^	-
**C/H ratio**	1.7^a^	13.2^a^	-
**Z-average (nm)**^d^			
**3.24 mg/ml**	625	271	112
**1.08 mg/ml**	251	252	-
**0.36 mg/ml**	199	250	-
**Polydisersity Index**^e^	0.540	0.339	0.219
**Zeta potential (mV)**^e^	-49.7	-13.9	-30.2
**pH**^e^	2.6	4.4	6.5

Lateral size, specific surface area, C/O and C/H ratios were determined using transmission electron microscopy (TEM), Brunauer–Emmet–Teller (BET) and combustion elemental analysis, respectively.

^a^ Adapted from Bengtson et al [26].

^b^ Size of spherical P90 particles based on surface area (338 m^2^/g) and density (2.1 g/m^3^) adapted from Saber et al [44]

^c^ Adapted from Jacobsen et al [48].

^d^ Mean hydrodynamic size (Z-average) were determined using Dynamic Light Scattering in 0.1% TW80.

^e^ Determined at particle concentration 3.24 mg/ml in 0.1% TW80.

As expected, zeta potential for rGO (-10.7 ± 0.6, pH = 4.4) indicated instability in vehicle, while GO (-39.3 ± 1.5, pH = 2.6) and P90 (-30.2 ± 0.2, pH = 6.5) were stable (S5 Fig). Hydrodynamic size, PDI, zeta-potential and pH for GO dispersed in PBS (0.36 μg/ml) was also determined (Size = 157 nm; PDI = 0.284; zeta potential = -24.7; pH = 6.9).

### Animal bodyweight and observations

The bodyweight of the instilled mice was monitored at 3–5 time points throughout the study period (S2 Table). In general, mice exposed to either VC, rGO or P90 increased their bodyweight at day 1 after instillation until day 90. In contrast, exposure to 54 or 162 μg/mouse of GO resulted in weight loss at day 1. The weight loss continued until day 3 with a total weight loss of ~15% and ~20% in bodyweight for the mice instilled with 54 and 162 μg/mouse, respectively. Signs of discomfort including energy loss, back arching and piloerection were observed. Further, the mice did not interact with each other. Due to animal welfare concerns based on the large weight loss and discomfort in groups of mice exposed to GO (54 and 162 μg/mouse), we immediately aborted the studyat day 3 and euthanized the mice in these groups. Thus, only groups exposed to GO 18 μg/mouse were included at days 28 and 90.

### Pulmonary inflammation

**Fig 1:**
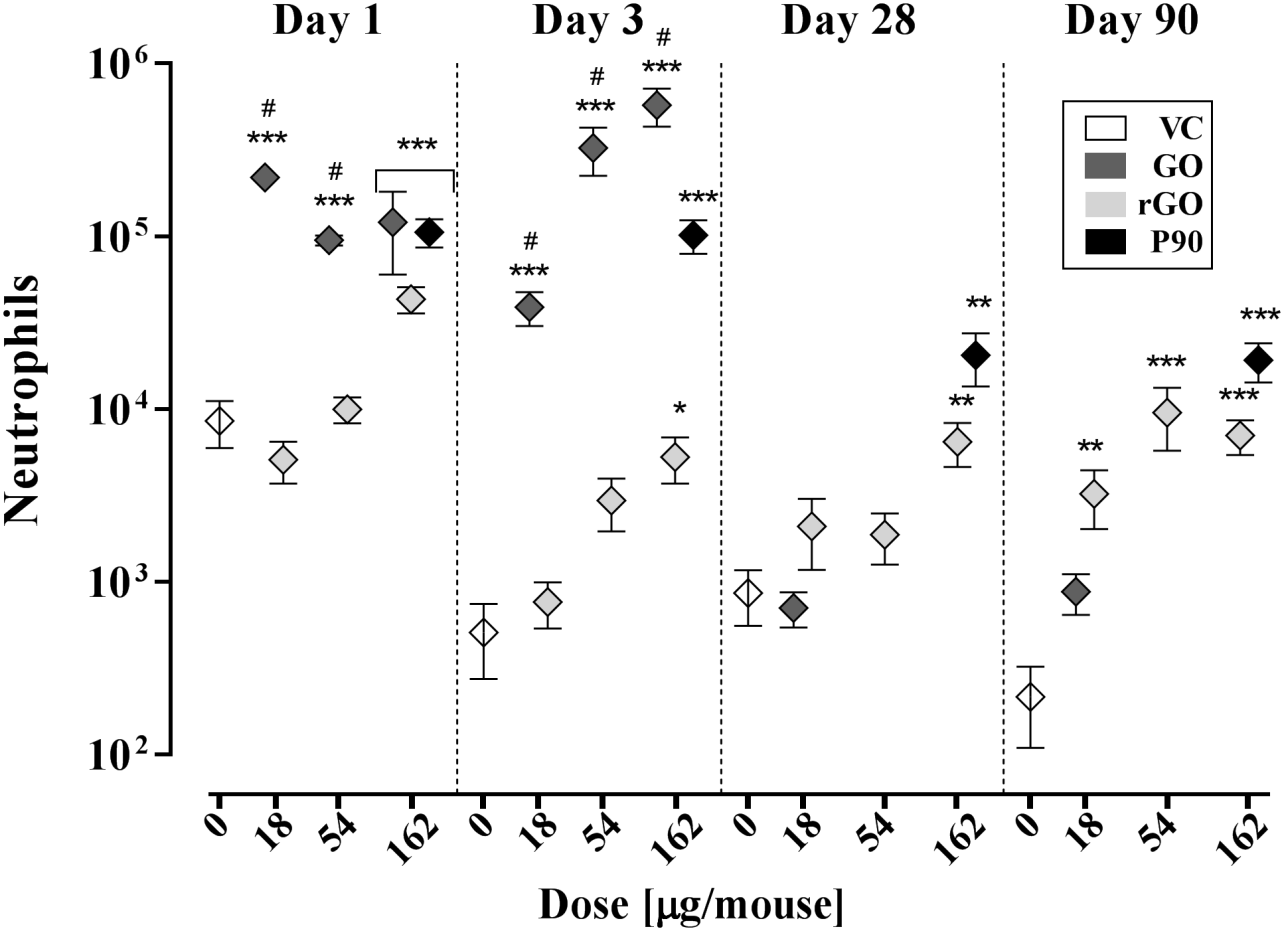
Graphical presentation of the number of neutrophils in bronchoalveolar lavage (Mean ± SEM) from at day 1, 3, 28 and 90 following exposure to VC, GO, rGO or P90 (*n* = 7–8). *, ** and ***: Statistically significantly different from corresponding VC at level *p* < 0.05, *p* < 0.01, *p* < 0.001, respectively. ^#^: GO statistically significantly different from corresponding rGO group at level *p* <0.001. For group GO 18 μg/mouse at day 1, the symbol is larger than the corresponding error bar. Therefore, the error bar is not visible (SEM is shown in Table 2).

Pulmonary inflammation was determined as number of neutrophil influx in BAL fluid. Cell differential counts in BAL fluid were determined on day 1, 3, 28 and 90. Overall, the level of inflammation differed across materials and doses (Fig 1 and Table 2).

**Table 2:** BAL fluid cell counts (x10^3^) (mean ±SEM) in mice at day 1, 3, 28 and 90 post exposure to VC (0.1% TW80), GO, rGO or Printex90 at doses 0, 18, 54 or 162 μg/mouse (*n* = 7–8).

	Dose	Neutrophils^a^	Macrophages^a^	Eosinophils	Lymphocytes	Total^a^
**Day 1**						
**VC**	**0**	8.5 ± 2.6	63 ± 19.4	0.2 ± 0.1	1 ± 0.8	72.6 ± 20.7
**GO**	**18**	219.5 ± 18.7***^#^	59.8 ± 5.6	5.7 ± 3.9	1.4 ± 0.9	294.7 ± 20.7***
	**54**	94.9 ± 6.4***^#^	29.1 ± 4.1*	0.5 ± 0	0 ± 0	123.9 ± 8.8***
	**162**	120.7 ± 60.6***	92.4 ± 8.5	96.2 ± 93.6	0 ± 0	309.3 ± 109.7***
**rGO**	**18**	5.1 ± 1.4	44.4 ± 9.2	0.5 ± 0.3	0.5 ± 0.2	20.2 ± 10.5
	**54**	10 ± 1.8	61.7 ± 7	1.4 ± 0.2	0.4 ± 0.2	73.5 ± 7.8
	**162**	43.3 ± 7.5***	68.7 ± 9.6	5.3 ± 2.5	0.1 ± 0.1	117.4 ± 16.1
**P90**	**162**	105.6 ± 19.5***	24.4 ± 9.4	5.5 ± 2.3	0 ± 0	135.6 ± 17.1*
**Day 3**						
**VC**	**0**	0.5 ± 0.2	32.9 ± 3.9	0 ± 0	0 ± 0	33.5 ± 3.9
**GO**	**18**	38.8 ± 8.6***^#^	78.7 ± 6***	51.3 ± 14.6	2.9 ± 1	171.7 ± 22.4***
	**54**	324.7 ± 100.8***^#^	171.2 ± 36***	10.8 ± 9.7	8.7 ± 2.2	515.5 ± 141.6***
	**162**	574.4 ± 142.9***^#^	149.6 ± 15.9***	0.6 ± 0.6	2.2 ± 1.4	727 ± 158.8***
**rGO**	**18**	5.7 ± 4.9	43.2 ± 5.1	12.5 ± 12.2	0.9 ± 0.8	62.2 ± 21.9
	**54**	3 ± 1	49.4 ± 7.1	1.6 ± 0.4	0.3 ± 0.2	54.2 ± 7.9
	**162**	5.3 ± 1.6*	50.1 ± 7.1	11.6 ± 5.8	0.2 ± 0.1	67.1 ± 11.5*
**P90**	**162**	101.3 ± 22.3***	55.7 ± 7.2*	5.5 ± 2	0.8 ± 0.6	163.3 ± 26.4***
**Day 28**						
**VC**	**0**	0.9 ± 0.3	17.7 ± 5.4	0 ± 0	0.1 ± 0.1	18.7 ± 5.5
**GO**	**18**	0.7 ± 0.2	45 ± 2.8**	0 ± 0	1 ± 0.5	46.7 ± 2.9**
**rGO**	**18**	2.1 ± 0.9	53.1 ± 10.5*	3 ± 1.7	0.1 ± 0.1	58.3 ± 11.1
	**54**	1.9 ± 0.6	55 ± 5.6**	0.5 ± 0.3	0.3 ± 0.2	57.8 ± 5.6*
	**162**	6.5 ± 1.8	57.2 ± 10.2**	0 ± 0	0.5 ± 0.2	64.2 ± 11.2*
**P90**	**162**	20.4 ± 6.9**	78.9 ± 12.4**	0 ± 0	3.9 ± 1.5	103.2 ± 17.2**
**Day 90**						
**VC**	**0**	0.3 ± 0.1	43.3 ± 5	0 ± 0	0.1 ± 0	43.6 ± 5
**GO**	**18**	0.9 ± 0.2	43.7 ± 4.9	0 ± 0	0.4 ± 0.1	45 ± 5.1
**rGO**	**18**	3.2 ± 1.2**	37.3 ± 4.5	0.2 ± 0.1	0.4 ± 0.1	41.2 ± 5.7
	**54**	9.5 ± 3.8***	40.4 ± 7.5	0.1 ± 0.1	0.7 ± 0.3	50.6 ± 7.2
	**162**	7 ± 1.6***	48.9 ± 4	0 ± 0	0.4 ± 0.2	56.3 ± 4.6
**P90**	**162**	19.2 ± 4.9***	62.6 ± 5.5*	0.2 ± 0.1	4.2 ± 0.9	86.1 ± 9.5***

Mean ± SEM (*n* = 7–8).

^a^ Statistical analysis was performed on neutrophils, macrophages and total cells.

*, ** and ***: Statistically significantly different from corresponding VC at level *p* < 0.05, *p* < 0.01, *p* < 0.001 level, respectively.

^#^: Statistically significantly different from corresponding rGO group at level *p* <0.001.

#### Day1

GO induced a potent and significant neutrophil influx at all doses compared to VC but no dose-response relationship was observed. For rGO, 162 μg/mouse induced statistically significantly increased neutrophil influx compared to VC with dose-response relationship. For the lower doses (18 and 54 μg/mouse), the level of inflammation was significantly lower for rGO compared to GO. The reference material P90 induced statistically significant inflammation compared to VC

#### Day 3

Both materials induced inflammation at day 3. GO was consistently the most inflammogenic across all doses. GO induced statistically significantly increased neutrophil influx at all dose levels compared to VC. For rGO, only the 162 μg/mouse dose induced statistically significantly increased neutrophil influx compared to VC. In comparison, all rGO groups (18, 54 and 162 μg/mouse) induced statistically significantly lower neutrophil influx than for the corresponding GO groups. GO and rGO appeared very different in BAL fluid. GO was mainly observed as already phagocytosed particles in 18 μg/mouse group (data not shown) while both free and phagocytosed particles were observed in 162 μg/mouse group, where large agglomerates of GO particles covered the cells (S2 Fig). For rGO (162 μg/mouse), large and compact agglomerates of particles were observed together with particles phagocytosed by macrophages. The reference material P90 induced statistically significant inflammation compared to VC

#### Day 28 and 90

Statistically significantly increased neutrophil influx was observed at day 28 only for rGO (162 μg/mouse) compared to VC. At day 90, all doses of rGO induced statistically significantly increased neutrophil influx compared to VC. The neutrophil influx for GO 18 μg/mouse group was between the level for the VC group and the rGO 18 μg/mouse group and did not differ statistically significantly from either of the two groups. P90 induced statistically significant inflammation at both time points,

#### No effect of pH on pulmonary inflammation

GO was delivered as water-suspension and the pH of the instilled GO dispersed in 0.1% TW80 was 2.6. In order to assess the effect of pH, we compared the inflammatory response to GO when dispersed in 0.1% TW80 (pH = 2.6) or PBS (pH = 6.9), respectively (S2 Table). The lowest number of neutrophils was observed in GO_TW80_ group (67% of the level for GO_PBS_ group), indicating that the observed neutrophil influx for GO dispersed in 0.1% TW80 was not caused by a low pH.

### Acute phase response

#### *Saa3* mRNA expression in the lung

**Fig 2:**
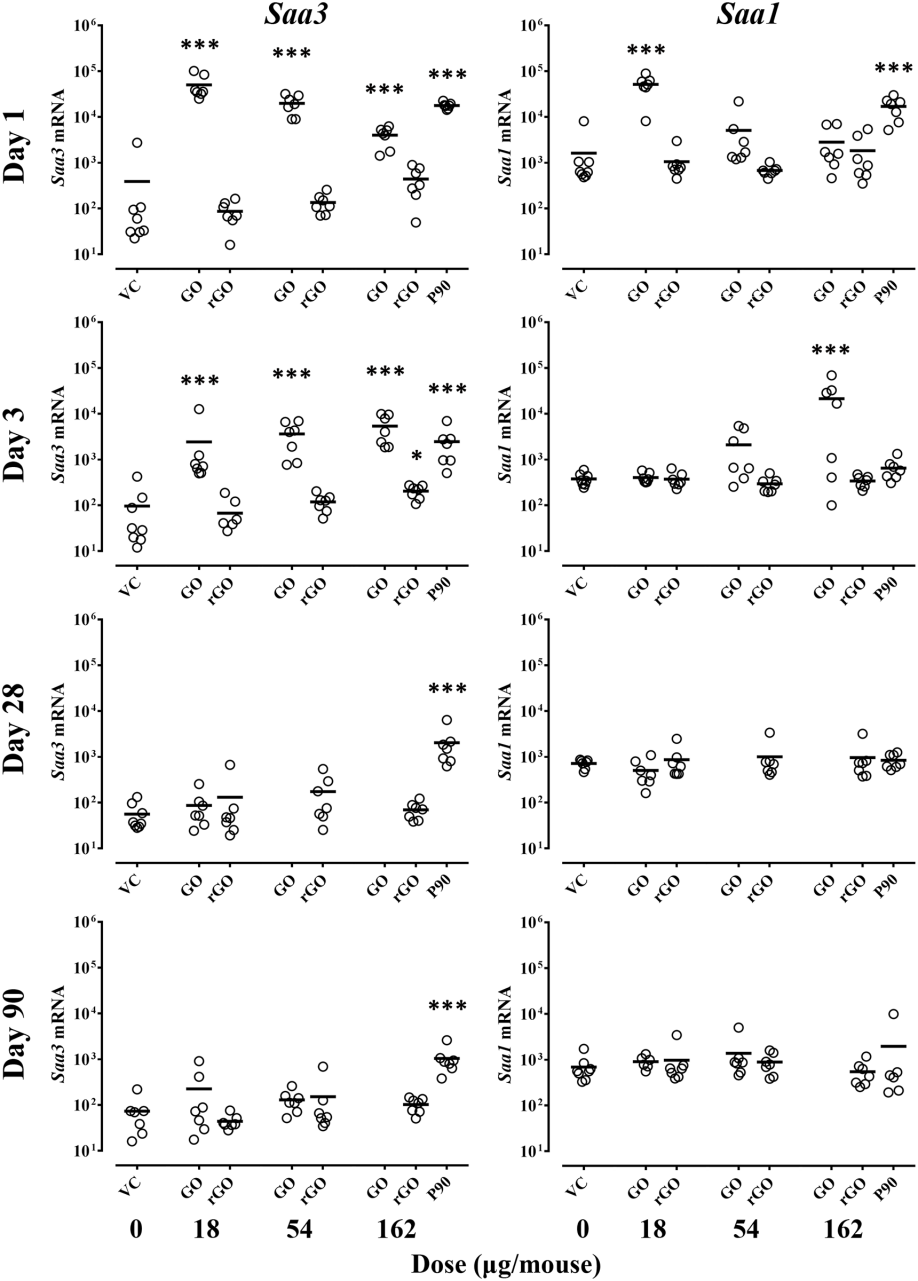
mRNA expression level of the acute phase response genes *Saa3* in lung and *Saa1* in liver 1, 3, 28 and 90 days following exposure to VC, GO, rGO and P90 (*n* = 7–8) at doses 0, 18, 54 and 162 μg/mouse. Expression of mRNA was normalized to 18S rRNA and multiplied by 10^7^. Statistical analysis for P90 compared to VC was performed separately using a t-test. *, **, ***: Statistically significantly different from corresponding VC at level *p* < 0.05, *p* < 0.01, *p* < 0.001, respectively.

Overall, GO was the strongest inducer of *Saa3* expression (Fig 2 and Table 3). At day 1, GO induced statistically significantly increased *Saa3* expression with 113, 45, and 9-fold increase compared to VC for 18, 54 and 162 μg/mouse, respectively. At day 3, the increase in mRNA expression levels of *Saa3* was also statistically significant compared to VC with increase in *Saa3* for 18, 54 and 162 μg/mouse (22, 33 and 49-fold increase respectively). In contrast, rGO induced only a small statistically significant increase in *Saa3* expression levels (2-fold) at day 3 compared to VC. At day 28 and day 90, no statistical significant increase in *Saa3* was observed for either GO or rGO compared to VC. For the reference material P90, a statistically significantly increased *Saa3* expression level was observed at all time points compared to VC. The association between *Saa3* mRNA levels and neutrophil influx in BAL fluid is shown in S6 Fig.

**Table 3:** mRNA expression level of *Saa3* and *Saa1* in lung and liver of mice 1, 3, 28 and 90 days post exposure to VC, GO, rGO or P90 at doses 0, 18, 54 or 162 μg/mouse.

		Day 1	Day 3	Day 28	Day 90
	Dose	*Saa3* mRNA			
**VC**	**0**	389 ± 335	96 ± 50	60 ± 15	74 ± 26
**GO**	**18**	50159 ± 11214 (128.9)***	2436 ± 1706 (25.4)***	80 ± 27 (1.3)	226 ± 125 (3.1)
	**54**	19795 ± 3443 (50.9)***	3631 ± 968 (37.8)***	-	-
	**162**	4016 ± 685 (10.3)***	5367 ± 1379 (55.9)***	-	-
**rGO**	**18**	87 ± 19 (0.2)	68 ± 24 (0.7)	131 ± 90 (2.2)	44 ± 6 (0.6)
	**54**	135 ± 25 (0.3)	119 ± 19 (1.2)	174 ± 72 (2.9)	152 ± 90 (2.1)
	**162**	441 ± 117 (1.1)	204 ± 24 (2.1)*	70 ± 11 (1.2)	102 ± 14 (1.4)
**P90**	**162**	17803 ± 1023 (45.8)***	2459 ± 828 (25.6)***	2036 ± 758 (33.9)***	1044 ± 274 (14.1)***
		***Saa1* mRNA**			
**VC**	**0**	1624 ± 927	377 ± 41	717 ± 58	689 ± 159
**GO**	**18**	51404 ± 9158 (31.7)*	403 ± 38 (1.1)	506 ± 124 (0.7)	906 ± 113 (1.3)
	**54**	5097 ± 2849 (3.1)	2102 ± 833 (5.6)	-	-
	**162**	2824 ± 1063 (1.7)	21287 ± 9518 (56.5)***	-	-
**rGO**	**18**	1057 ± 327 (0.7)	373 ± 53 (1)	869 ± 280 (1.2)	973 ± 416 (1.4)
	**54**	678 ± 68 (0.4)	294 ± 42 (0.8)	1001 ± 399 (1.4)	886 ± 175 (1.3)
	**162**	1839 ± 753 (1.1)	340 ± 36 (0.9)	964 ± 377 (1.3)	546 ± 124 (0.8)
**P90**	**162**	16956 ± 3334 (10.4)***	649 ± 130 (1.7)	840 ± 113 (1.2)	1963 ± 1603 (2.8)

Mean ± SEM (fold change), *n* = 7–8

mRNA was normalized to 18S rRNA and multiplied by 107.

*, **, ***: statistically significantly different from corresponding VC at level *p* < 0.05, *p* < 0.01, *p* < 0.001, respectively.

#### *Saa1* mRNA expression in the liver

Overall, only GO induced statistically significantly increased *Saa1* mRNA expression compared to VC (Fig 2 and Table 3) compared to VC. At day 1, GO induced the highest *Saa1* mRNA expression (18 μg/mouse). At day 3, we observed statistically significant increased *Saa1* expression for the highest dose of GO (162 μg/mouse) compared to VC. At day 28 and day 90, no statistically significantly increased *Saa1* expression was observed. P90 induced statistically significantly increased *Saa1* expression only at day 1 when compared to VC.

#### SAA3 in blood

At day 3, we observed statistically significantly increased SAA3 in blood for groups exposed to GO (54 and 162 μg/mouse) compared to VC. rGO exposure did not affect SAA3 levels (S7 Fig).

### Histopathological analysis

**Fig 3:**
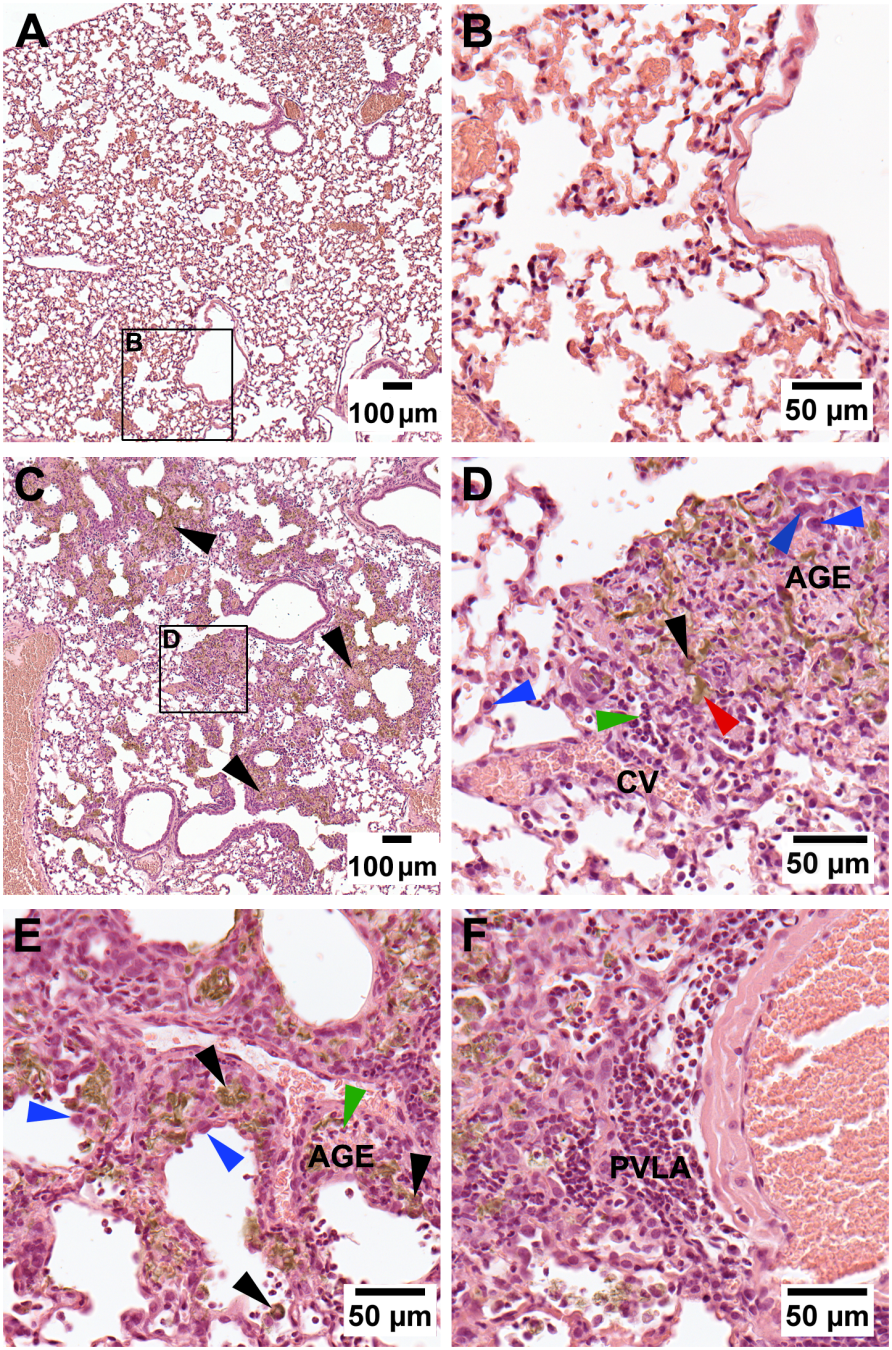
H&E stained histopathological lung sections of mice 3 days post exposure. VC (A, B) and GO 162 μg/mouse (C, D, E, F). (A and B) No pathological changes. (C) Patchy appearance of acute pulmonary inflammation in areas with GO deposits (black arrows) in the parenchyma distal to the terminal and respiratory bronchioles, alveolar ducts, alveoli. (D) Free GO deposits (red arrow) and within alveolar cells (black arrows) in inflammatory area. Accumulation of granulocytes (green arrow). Hyperplastic type II cells (blue arrow). Congestion of vessels (CV). Alveolar granular exudate (AGE). (E) Patchy inflammation in peripheral section sites with GO deposits. Alveolar macrophage with GO in inflammatory lesion and in alveoli (black arrows), polymorphonuclear leukocytes (green arrow). Alveolar granular exudate (AGE). Hyperplastic type II cells (blue arrows). (F) Perivascular lymphoid accumulation (PVLA) with GO deposits.

Due to the high acute toxicity of GO, day 3 was included in the histopathological analysis of lung tissue in addition to day 90. At day 3, GO was observed as light brownish granular pigments (Fig 3c–3f and S8 Fig). Granulocytes and macrophages with or without GO were mostly seen in the alveolar walls followed by alveolar sacs and alveolar ducts, and to some extent in the terminal bronchioles including adjacent interstitium. Observations indicated severe acute inflammatory response, involving air passages and respiratory segments distal to the ciliated airways. GO was more prominent and visible in the 162 μg/mouse group compared to 54 μg/mouse group, and was observed mainly as free deposits or in aggregates in macrophages within the alveoli and the alveolar ducts. In addition, inflammation at sites with GO deposits was prominent and patchy (Fig 3c) and located to the respiratory parenchyma with a larger total area affected with increased dose (~ 20–30% and 30–40% of the total cut lung surface for groups 54 and162 μg/mouse, respectively). The alveolar walls were enlarged and neutrophils were found together with congested capillaries and alveolar granular exudate as part of the acute inflammation (Fig 3d and 3e). Hyperplastic Type II cells were also observed (Fig 3d and 3e). These pathological findings were notably found in group 162 μg/mouse as part of the acute inflammation. In general, areas devoid of dusts were mainly without pathological findings. Perivascular lymphocytic accumulations were also clearly observed (Fig 3f).

**Fig 4:**
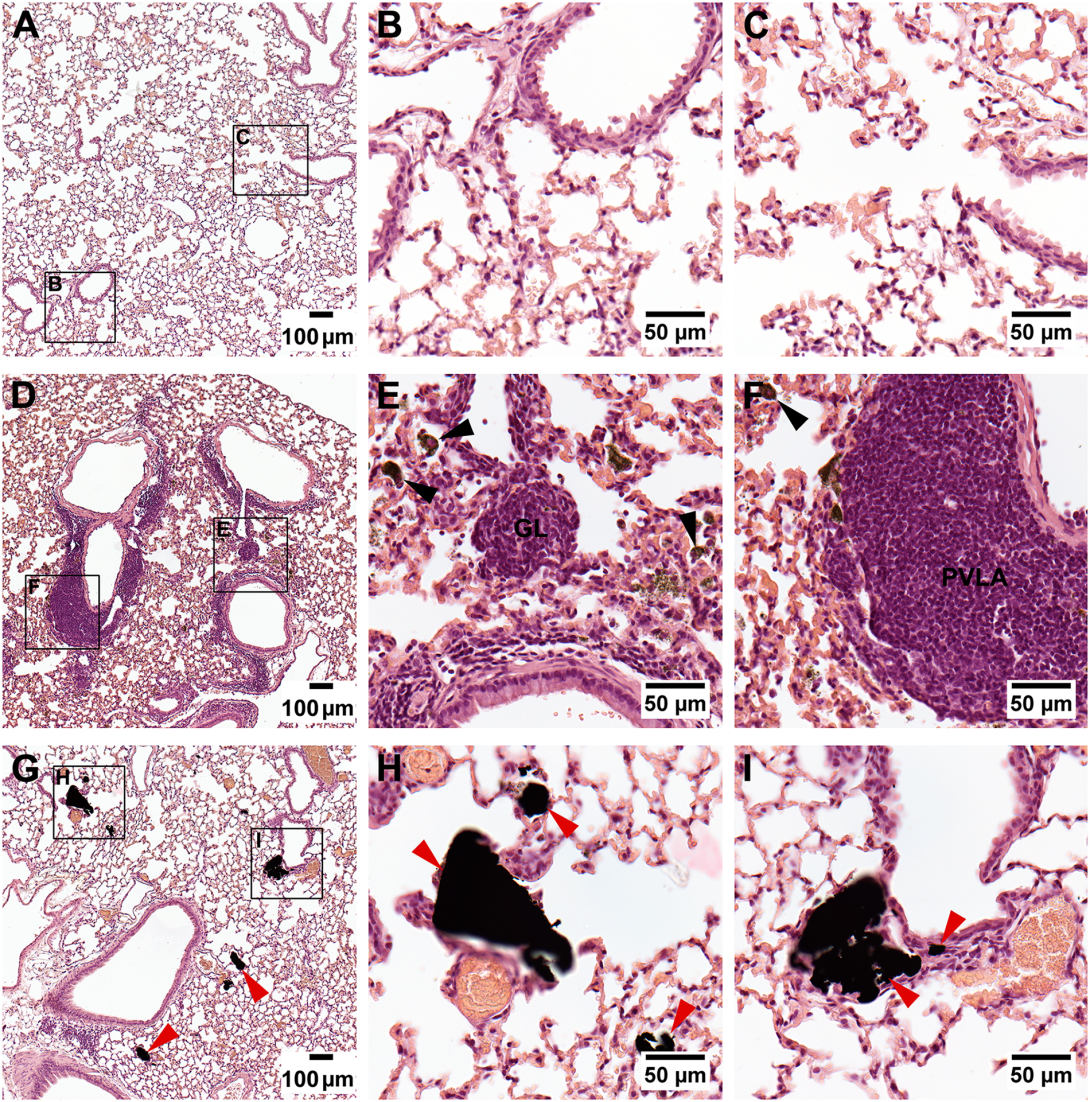
H&E stained histopathological lung sections of mice 90 days post exposure. VC (A-C), GO 18 μg/mouse (D-F) or rGO 162 μg/mouse (G-I). (A-C) No pathological changes. (D-F) GO appeared as dark-brown pigments. Scattered prominent perivascular lymphoid accumulation (PVLA). Granuloma formation (GL) containing GO and macrophages with GO in alveoli (black arrows). Rare prominent perivascular lymphocytic accumulation (AGE). (G-I) Scarce accumulation of compact black rGO agglomerates (red arrows) and minimal tissue reactions.

At day90, GO appeared as smaller and more brown pigments. Fewer macrophages and complete engulfment of GO in macrophages were also observed (Fig 4e). Remnants from the acute inflammatory response were minimal and observed mainly as small spots with chronic inflammation in alveolar walls affecting approximately 2–4% of the total examined lung section surface area (Fig 4d and 4e). Chronic inflammatory cells loaded with GO in the alveolar walls were observed (~ 2–4% of the cut lung surface). Perivascular lymphocytic accumulations were also observed (Fig 4f). Birefringent collagen was not observed and there were no findings indicating lung fibrosis.

In the histopathological analysis of rGO at day 90, rGO appeared as black compact agglomerated deposits. Agglomerates were observed with focal distribution in the peripheral lung parenchyma, lumen of terminal bronchioles, alveolar ducts and in the alveolar septal walls (Fig 4g–4i). The largest rGO agglomerates were 4–6 times larger than alveolar macrophages and only a minor fraction of rGO were phagocytosed. Agglomerates deposited in the bronchioles, more frequently in group 162 μg/mouse, may represent incompletely dispersed rGO either trapped in the peripheral airways or in process of being cleared. Taken together, few tissue reactions were observed in the high dose group and only very few in the low dose groups. Areas with slightly increased cells in alveolar walls with dusts were not numerous. Some minor dust-related granulomas and minimal increased septal cellularity were observed in GO-exposed group. Birefringent collagen was not identified and there were no findings indicating lung fibrosis. It should be noted that specific stains for collagen-formation was not included in the histopathology analysis.

### Genotoxicity

DNA damage were assessed in BAL cells, lung and liver tissue. GO and rGO induced statistically significantly increased level of DNA damage in BAL cells across particles and doses at different time points (Table 4). GO (18 μg/mouse) induced increased levels of DNA damage in BAL cells at day 3 and 28 compared to VC. rGO (18 μg/mouse) induced increased DNA damage at day 28 and at day 90 compared to VC. No statistically significantly increased DNA damage was observed for GO and rGO in lung and liver compared to VC. P90 induced increased level of DNA damage in liver at day 90.

**Table 4:** DNA strand breaks. Level of DNA damage (Mean ± SEM) in BAL, lung and liver assessed with the comet assay (% DNA) 1, 3, 28 and 90 days post exposure to VC, GO, rGO or P90 (*n* = 7–8).

		Day 1	Day 3	Day 28	Day 90
	Dose	BAL			
**VC**	**0**	0.44 ± 0.06	0.60 ± 0.04	1.19 ± 0.17	1.00 ± 0.11
**GO**	**18**	0.61 ± 0.03	0.98 ± 0.04*	1.87 ± 0.15*	1.18 ± 0.13
	**54**	0.54 ± 0.03	0.68 ± 0.03	-	-
	**162**	0.55 ± 0.07	0.51 ± 0.06	-	-
**rGO**	**18**	0.66 ± 0.06*	0.60 ± 0.05	1.57 ± 0.20	1.73 ± 0.21*
	**54**	0.65 ± 0.04*	0.68 ± 0.07	1.01 ± 0.13	1.27 ± 0.11
	**162**	0.48 ± 0.03	0.74 ± 0.07	1.33 ± 0.11	1.55 ± 0.14
**P90**	**162**	0.39 ± 0.02	0.75 ± 0.02**	0.87 ± 0.06	0.81 ± 0.05
		**Lung**			
**VC**	**0**	0.54 ± 0.02	0.97 ± 0.08	1.47 ± 0.12	2.05 ± 0.16
**GO**	**18**	0.46 ± 0.04	0.99 ± 0.07	1.67 ± 0.18	1.64 ± 0.13
	**54**	0.42 ± 0.03	1.09 ± 0.06	-	-
	**162**	0.43 ± 0.04	1.21 ± 0.14	-	-
**rGO**	**18**	0.56 ± 0.10	0.79 ± 0.07	1.57 ± 0.17	1.80 ± 0.28
	**54**	0.87 ± 0.17	0.92 ± 0.07	1.73 ± 0.23	1.88 ± 0.24
	**162**	0.63 ± 0.08	1.07 ± 0.11	1.60 ± 0.13	1.58 ± 0.23
**P90**	**162**	0.78 ± 0.12	1.04 ± 0.07	1.58 ± 0.08	2.15 ± 0.19
		**Liver**			
**VC**	**0**	1.75 ± 0.12	1.56 ± 0.21	1.04 ± 0.10	0.66 ± 0.03
**GO**	**18**	1.71 ± 0.30	1.62 ± 0.13	0.87 ± 0.09	0.72 ± 0.03
	**54**	2.06 ± 0.20	1.92 ± 0.26	-	-
	**162**	1.56 ± 0.22	1.65 ± 0.15	-	-
**rGO**	**18**	1.49 ± 0.19	1.31 ± 0.06	0.83 ± 0.08	0.69 ± 0.08
	**54**	1.34 ± 0.20	1.41 ± 0.16	0.98 ± 0.14	0.66 ± 0.06
	**162**	1.46 ± 0.22	1.71 ± 0.19	0.91 ± 0.05	0.83 ± 0.15
**P90**	**162**	1.43 ± 0.19	1.62 ± 0.19	0.80 ± 0.10	0.91 ± 0.04**

Data were normalized to the mean level of %DNA of H_2_O_2_-exposed A549 cells included on each slide during each electrophoresis (S3 Fig). All samples from BAL, lung and liver were divided onto different electrophoresis and further divided according to time point post exposure to minimize day-to-day variation.

*, **, ***: statistically significantly different from corresponding VC at level *p* < 0.05, *p* < 0.01, *p* < 0.001, respectively.

### Work place exposure to graphene

During the chemical vapour deposition process at case 1, opening the reactor in the beginning of the process, did not cause any measurable emissions. The same applied when the reactor was turned on. During the reactor opening after the process, the CPC showed a small increase in NF particle concentration (< 20 particles/cm^3^) that was not detected by the DiSCmini. Cleaning of the chamber was done after the process and then particle concentration rose to 15 particles/cm^3^ (S9 Fig). Analysis of the TEM-grids sampled showed no sign of particle presence. Case 2 differed from the first case by not being a clean room location which made it more complicated to estimate possible emissions solely relating to the process run due to a high presence of background particles. Generally, the NF and FF CPC concentrations had the same trends, with NF being a bit higher. The chemical vapour deposition process-related activities did not have a significant effect on the aerosol number concentrations (S10 Fig). Different kind of process-related events were performed during measurements, but no link between activity and concentration could be observed. Analyzed TEM-grids that were sampled upon during the opening of the reactor showed traces of amorphous carbon compounds deposited onto them (data not shown). However, the same compounds were observed on grids sampled elsewhere within the room.

## Discussion

This current study showed that pulmonary deposition of GO or rGO, comparable in layer numbers (2–3 layers) and lateral size (> 1–2 um), but with different levels of hydroxylation, induced pulmonary inflammation. Overall, GO was much more inflammogenic than rGO at day 1 and 3 whereas rGO induced sustained inflammation for up to 90 days post exposure with no apparent decrease from day 3. Due to the discomfort and weight loss of mice following exposure to higher doses of GO, only the lowest dose group was followed for beyond day 3.

The potent acute inflammatory response caused by GO peaking at day 3 was accompanied by a strong and transient increase in pulmonary and hepatic mRNA levels of APR genes (*Saa3* and *Saa1*, respectively), that was shown to be systemic for SAA3. These findings were supported by histopathological analyses of lung sections showing severe acute inflammation for GO at day 3. At day 90, GO agglomerates (larger for rGO) were still present in the lungs, though only minimal inflammation and no sign of fibrosis were observed. Both materials were also found to increase the level of DNA damage in BAL cells across doses and time points.

There are currently only a limited number of *in vivo* studies investigating toxicity following pulmonary exposure to well-characterized graphene-based materials. However, due to the physicochemical properties of GO for use in a broad range of biomedical applications [34,41,57], the number of published studies evaluating toxicity of GO is increasing. GO has previously been shown to be very biopersistent, causing both a severe and delayed peak in pulmonary inflammation, followed by chronic inflammation up to 3 months after exposure [36]. In another study, GO induced disruptions of the alveolar-capillary barrier accompanying influx of neutrophils into the alveolar space and release of inflammatory cytokines after 24 hours [35]. The same study [35] also showed that GO is more inflammogenic than pristine graphene.

We observed a clear difference in color and dispersion of GO (brown colored) and rGO in vehicle (black colored). rGO formed agglomerates that are caused by the high hydrophobicity and colloid instability in aqueous solutions, that is well known [58–60]. Aggregate formation of graphene has been shown to increase toxicity [35]. As vehicle, we chose to use 0.1% TW80, which has been shown not to trigger an acute inflammation in mice [61–64]. TW80 improved the stability of rGO and allowed an accurate and reproducible dose delivery by instillation procedure within 30 minutes after sonication. Though a complete colloid stability was never achieved, this study confirmed that intratracheal instillation of 0.1% TW80 did not increase number of neutrophils compared to PBS as vehicle.

Instillation is generally recognized as an acceptable exposure technique that offers precise dosing and even particle distribution in the lung [25,65]. Advantages and disadvantages of instillation have been discussed previously [66,67]. We used dose levels that equal pulmonary deposition for 1, 3, and 9 days (8 hours/day) at the current Danish occupational exposure limit of 3.5 mg/m^3^ Carbon Black per 8 hour work shift [16] or 1, 3 and 9 times the expected exposure for workers (40 years) at the recommended exposure limit for CNT of 0.001 μg/m^3^ per 8 hour work shift proposed by NIOSH [68]. We have published several studies using single instillation in mice as an exposure method (6–162 μg/mouse) for toxicity testing of carbon-based materials with different physicochemical properties and relatively free of inorganic impurities and endotoxin with similar dosing levels [16,25,44,69].

GO induced pulmonary inflammation at day 1. All doses resulted in what appeared as the maximum level of inflammation achievable 1 day post exposure. However, GO induced a delayed and very strong inflammation that peaked at day 3. We have previously observed a similar strong inflammation at day 1 followed by a peak at day 3 after instillation of MWCNT [19,23]. On the contrary, rGO-induced inflammation that peaked at day 1. This is very similar to the patterns observed for the spherical particles P90 and DEP [16,21,22].

The observed high acute toxicity of GO peaking at day 3 was further reflected in the decrease in bodyweight and signs of discomfort for mice exposed to 54 and 162 μg/mouse. Therefore, due to animal welfare concerns, the study only continued with low dose of GO (18 μg/mouse) at day 28 and 90. Findings are consistent with the current literature using mice as *in vivo* model. Li et al [36] found that instillation of 5–10 mg/kg (~100 or 200 μg/mouse) GO caused significant weight loss day 1 post exposure continuing until day 2 with total weight loss of ~20%. Using intravenous exposure route, another study reported a trend of sustained lower bodyweight compared to vehicle-exposed group following single intravenous injection of GO (~ 6 μg/mouse) [70]. Recently, a similar trend was also found following intravenous injection of 5 mg/kg GO (~ 100 μg/mouse) included lethal effect up to 40% 1 day post injection [71].

The rGO-induced neutrophil influx was consistently lower than the neutrophil influx induced by the reference P90 (162 μg/mouse). The very different pulmonary responses to GO and rGO may indicate that high level of hydroxyl-functionalization (O and H) found in GO is an important determinant of acute inflammation of graphene materials. Notably, rGO induced increased neutrophil influx 90 days post exposure.

Increased blood level of APR proteins has been recognized as risk a factor for cardiovascular disease [12,14,72]. SAA may be causally related to atherosclerosis and risk of cardiovascular disease. A biomonitoring study recently found correlation between dust exposure and blood levels of SAA and C-reactive protein [73]. We have previously proposed that pulmonary exposure to nanoparticles could trigger pulmonary APR with subsequent release of acute phase proteins e.g. SAA3 into the bloodstream [10,74]. In the blood, SAA is incorporated into HDL lipoproteins, replacing Apo A-1. SAA-HDL inhibits reverse cholesterol transport. It has been demonstrated that SAA stimulates cholesterol transport from lipoprotein to macrophages, thus stimulating macrophages to turn into foam cells [75]. In support of this notion, virus-mediated overexpression of SAA1 accelerates plaque progression in APOE -/- mice [76] and repeated pulmonary dosing of SAA1 does the same (Daniel V. Christophersen et al, unpublished). We have shown that P90 can induce sustained inflammation but only a transient pulmonary APR at a relatively low dose (18 μg/mouse) and sustained APR at higher dose (162 μg/mouse) [16,21]. Furthermore, we have also shown that standard diesel exhaust particle NIST1650b only induce a transient inflammatory and pulmonary APR [22]. Latest, we have shown that MWCNT can induce both pulmonary and hepatic APR at day 1 and 3 post exposure [23,24,74]. Together with strong pulmonary inflammation, GO induced both a transient pulmonary and hepatic APR at day 1 and 3. An increased level of SAA3 was also found in the blood at day 3. This suggests that GO can induce an acute APR that is very similar to MWCNT, that could also be the cause of the discomfort and the weight loss. We speculate which of the physicochemical properties of GO, but absent for rGO and P90, result in the strong APR. It was clearly not caused by the low pH (pH = 2.6) of the GO suspension, since GO dispersed in PBS (pH = 6.9) gave even stronger neutrophil influx, which correlates with APR. Moreover, it was not caused by organic and inorganic impurities since only very low levels were present [26]. An explanation could be the structural properties of GO. GO has been demonstrated to express a lower bending stiffness than pristine graphene [77,78]. We speculate whether the bending structure of GO may trigger toll-like receptor activation or whether GO with the high level of hydroxylation trigger similar responses as lipopolysaccharide.

Histopathological analysis conducted at day 3 revealed large focal deposits of GO in the lung parenchyma. This was accompanied by high deposition of GO that induced a severe inflammation with a trend of dose-dependency, from the ciliated airways to the alveoli region causing enlarged alveolar walls filled with neutrophils and edema.

Consistent with the BAL findings, the depositions and inflammatory responses were more pronounced in the highest exposure group. At day 90 GO (group 18 ug/mouse) and rGO (groups 54 and 162 ug/animal)were still present in lungs at day 90although neither thickening of the alveolar walls nor fibrosis formation were observed. Duch et al. [35] showed that well-dispersed graphene and GO (50 μg/mouse) did not induce fibrosis, although aggregated graphene did. Similar findings for rGO materials showing only little or no sign of fibrosis formation have been reported [6,7,27,35,79]. Observations in this study support previous findings, suggesting that graphene does not promote histopathological lung fibrosis, although particles are still found in the lung.

P90, DEP and MWCNT have all been shown to generate reactive oxygen species (ROS) in acellular assays [19,26,28,48]. Recently, we showed that GO generated more ROS compared to rGO but did not induce genotoxicity in murine lung epithelial cells *in vitro* [26]. The present study is the first to demonstrate that pulmonary exposure to these materials can induce DNA damage *in vivo* up to 90 days post exposure. We observed increased levels of DNA damage across doses and time points. These results are consistent with our previous studies in mice with similar experimental setup showing increased genotoxicity of P90 and DEP [16,21,22]. For MWCNT, the diameter was found to be a predictor, whereas the functionalization was not [25]. Results from the present study showed that increased levels of DNA damage were similar across GO and rGO. This shows that hydroxylation and particle-generated ROS by GO was not the main mechanism for genotoxicity.

In summary, pulmonary exposure to GO and rGO induced different pulmonary inflammatory responses in BAL. GO induced a strong acute response whereas rGO was much less inflammogenic. For rGO, pulmonary exposure induced long lasting inflammation even at the lowest assessed dose. However, only minimal acute inflammation was observed in lung tissue at day 90. Both materials induced DNA strand breaks long after exposure, indicating that the genotoxicity and inflammation were determined by different physicochemical properties of the graphene materials.

The production of graphene, produced by chemical vapour deposition, will increase although a lack of an industrial market has slowed down the progress in production capacity [41]. In this study, exposure measurement was performed at production facilities growing graphene on substrates in a fully closed reactor. Although events during production based on whole day measurement increased dust exposure temporarily, the number of particles was very low. Moreover, characterization of the particles confirmed a low level of graphene emission from the reactor. It is not unlikely that the small increase in particle number was due to disturbances of tubing and flows to the instrument during the opening and cleaning. In case 2, an increase in particle concentration was observed during the reactor warm-up, but since the increase is equally visible in the NF and FF, it is concluded to not be process related. Short peaks can be observed in the DiSCmini data which are not appearing in the CPC data, and these are suspected to be false concentrations due to influence of large particles in the DiSCmini detection system. Similar findings were reported by Fonseca et al. [80], where the uncertainties linked to the data provided by online particle instruments during quantitative exposure assessment levels to CNTs during conductive thin film production by chemical vapour deposition, were mainly due to high background concentration. The presence of a particulate background may hide a number-wise small emission, and thus it can only be concluded that no major emission of particulate matter took place during the process.

While the sampled TEM-grids did contain carbonaceous material, the amorphous nature of these makes it unlikely that it was actually graphene. Furthermore, similar material was collected on TEM-grids sampled further away from the chamber in the room. In general, the fact that the chamber volume was operating at a low pressure and was vented before being opened, means that the potential for exposure to airborne matter was very low. Heitbrink [42] assessed exposure to graphene at a normal production facility. Graphene was produced in steel containers where e.g. during cleaning of the containers, workers opened the hatch and used a hand tool to scrape graphene powder off the inside. This process created dust exposure. Our study showed that exposure to graphene can be minimized at workplaces during production in sealed chambers.

## Conclusion

Intratracheal instillation of GO and rGO in mice induced pulmonary inflammation. GO induced much more acute inflammation than rGO and induced a strong pulmonary and hepatic acute phase response. rGO induced inflammation that decreased from day 1 to 3 and then remained low but constantly elevated at a similar level until the end of the experiment at day 90. Further, rGO only induced minimal pulmonary, but no hepatic APR. Both materials were present in lung histopathological sections at day 90, although only minor inflammation was observed and there was no sign of fibrosis formation. Both materials were found to induce genotoxicity in BAL cells across doses and time points.

Work place measurements during graphene production using chemical vapour deposition showed no measurable risk of exposure to airborne graphene at the studied production sites.

## Supporting information

S1 Fig Schematic overview of exposure measurement setup at two different production sites. (a) in clean room and (b) at industrial site.
(TIF)

S2 Fig Representative May-Grünwald-Giemsa stained BAL cells. Difference in cell composition and deposition of graphene material present at day 3 post exposure to (A) VC, (B) GO 162 μg/mouse, (C) rGO 162 μg/mouse.
(TIF)

S3 Fig Day-to-day variation in level of DNA damage (%DNA) in the comet assay. A549 cells exposed to either PBS or H_2_O_2_ (60 μM) were included in each electrophoresis and thus used as internal electrophoresis controls in order to check day-to-day variation. Due to variation across each electrophoresis, all BAL, lung and liver samples were normalized by the value of PBS-exposed cells from the corresponding electrophoresis. Black lines denote mean values.
(TIF)

S4 Fig Hydrodynamic size distribution of GO, rGO and P90 dispersed in 0.1% TW80. Measurements were conducted using DLS and results are presented as percent intensity (left) and number (right) at 3.24 mg/ml (black solid lines), 1.08 mg/ml (grey dashed lines) and 0.36 mg/ml (black dotted lines), respectively.
(TIF)

S5 Fig Stability of GO and rGO in different suspensions. (A) rGO (3.24 mg/ml) was prepared in water added TW80 (1%, 0.1%, 0.01% or 0.001%) to visualize the effect on sedimentation. GO added 0.1% Tween80 (B) or PBS (C) that were used in this study (0.36 mg/ml). All suspensions were sonicated for 16 minutes, as described. In general, photos were captured within 30 minutes after sonication to reflect the time used to conduct the intratracheal instillation in mice.
(TIF)

S6 Fig Association between the pulmonary mRNA level of the acute phase response gene *Saa3* and total number of neutrophils in BAL fluid. Each dot represents an individual mouse (*n* = 195) exposed to either VC (white), GO (red), rGO (blue) or P90 (black). Color intensity denotes dose levels (18, 54 or 162 μg/mouse), where darker colored dots denotes higher levels. Triangles, diamonds, circles and squares denotes day 1, 3, 28 and 90, respectively.
(TIF)

S7 Fig SAA3 blood concentration 3 days post exposure to VC, GO or rGO. Samples in each group were pooled randomly to a final *n* = 3 (representing 6 samples). Black lines denote mean values.*, ** and ***: Statistically significantly different from VC at *p* < 0.05, *p* < 0.01, *p* < 0.001 level, respectively.
(TIF)

S8 Fig Acute inflammation 3 days post exposure to GO (162 μg/mouse). (A) Patchy appearance of acute pulmonary inflammation in areas with GO deposits (black arrows). (B) GO appeared as free light-brown granular pigments (red arrows).
(TIF)

S9 Fig Particle Number concentrations measured during graphene production in clean room. Measurements were conducted with CPC and DiSCmini in Near Field (NF) and Far Field (FF) during a work day with graphene production using chemical vapour deposition. In-graph numbers refers to time events: (1) Open reactor, (2) Close reactor, (3) Initiate growth, (4) Open reactor, (5) Dry wiping the reactor.
(TIF)

S10 Fig Particle Number concentrations measured during graphene production at industrial site. Measurements were conducted with CPC and DiSCmini in Near Field (NF) and Far Field (FF) during a work day with graphene production using chemical vapour deposition. In-graph numbers refers to time events: (1) Reactor Warm-up, (2) Open reactor, (3) Initiate growth, (4) Open nearby CNT chamber, (5) Open reactor–Wafer out, (6) Open reactor–Wafer out, (7) Open reactor–Wafer out.
(TIF)

S1 Table Bodyweight. Continuous bodyweight (g) measurements of mice exposed to VC, GO, rGO or P90.
(DOCX)

S2 Table BAL cell counts. Differential BAL cell count (x10^3^) from mice at 3 post exposure to 0.1% TW80, 18 μg/mouse GO in 0.1% TW80, 0.1% PBS or 18 μg/mouse GO in PBS.
(DOCX)
