# Supplementary figures and images for: Differences in inflammation and acute phase response but similar genotoxicity in mice following pulmonary exposure to graphene oxide and reduced graphene oxide
Source: PLoS One. 2017 Jun 1;12(6):e0178355. doi: 10.1371/journal.pone.0178355 (PMC5453440; doi:10.1371/journal.pone.0178355)

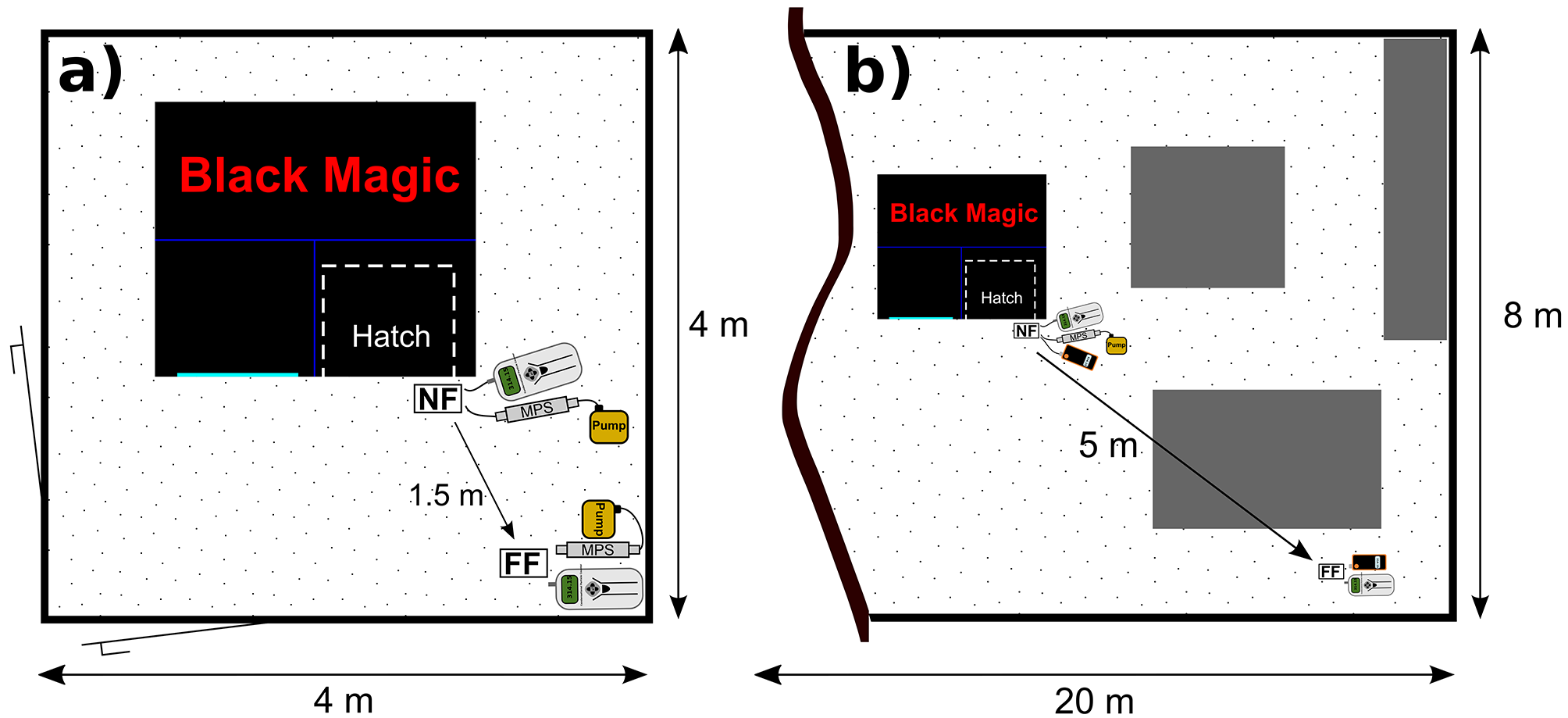

Supplement: S1 Fig — (a) in clean room and (b) at industrial site. (TIF) [file pone.0178355.s001.tif]

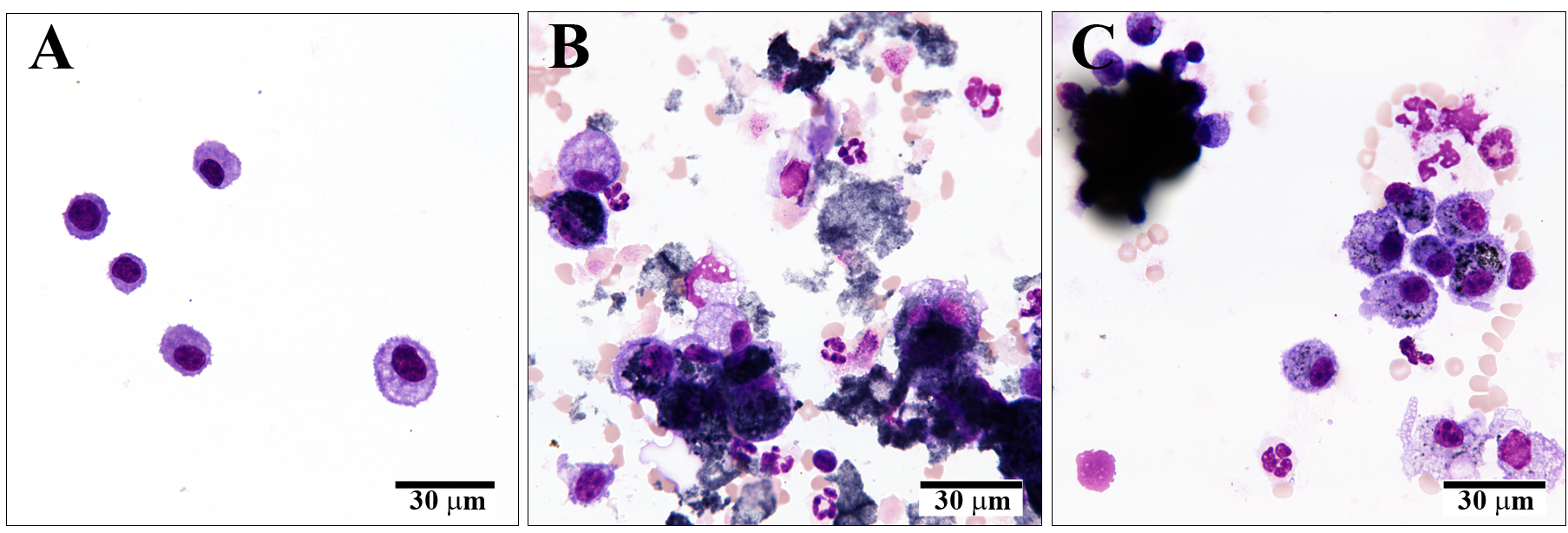

Supplement: S2 Fig — Difference in cell composition and deposition of graphene material present at day 3 post exposure to (A) VC, (B) GO 162 μg/mouse, (C) rGO 162 μg/mouse. (TIF) [file pone.0178355.s002.tif]

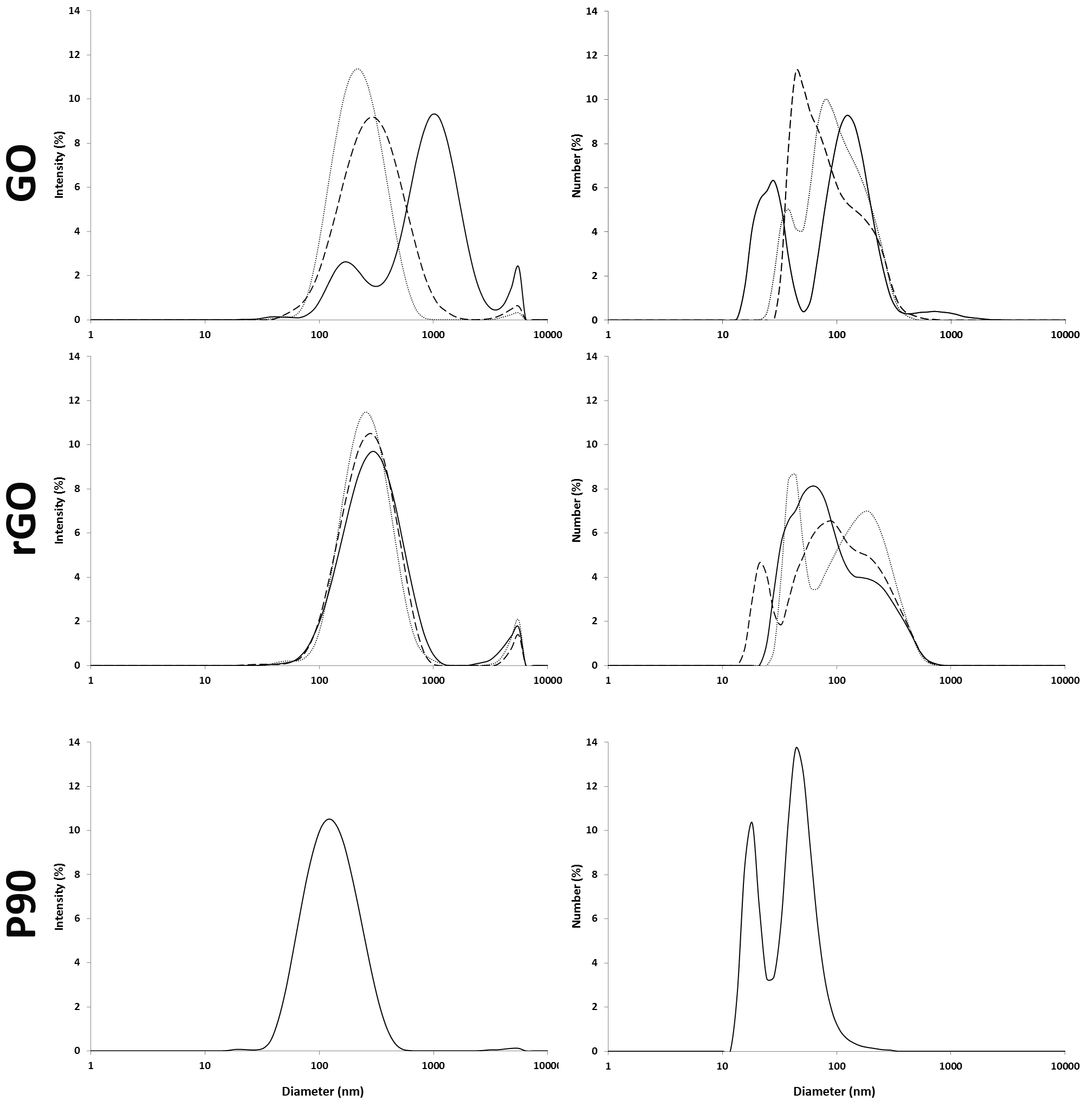

Supplement: S4 Fig — Measurements were conducted using DLS and results are presented as percent intensity (left) and number (right) at 3.24 mg/ml (black solid lines), 1.08 mg/ml (grey dashed lines) and 0.36 mg/ml (black dotted lines), respectively. (TIF) [file pone.0178355.s004.tif]

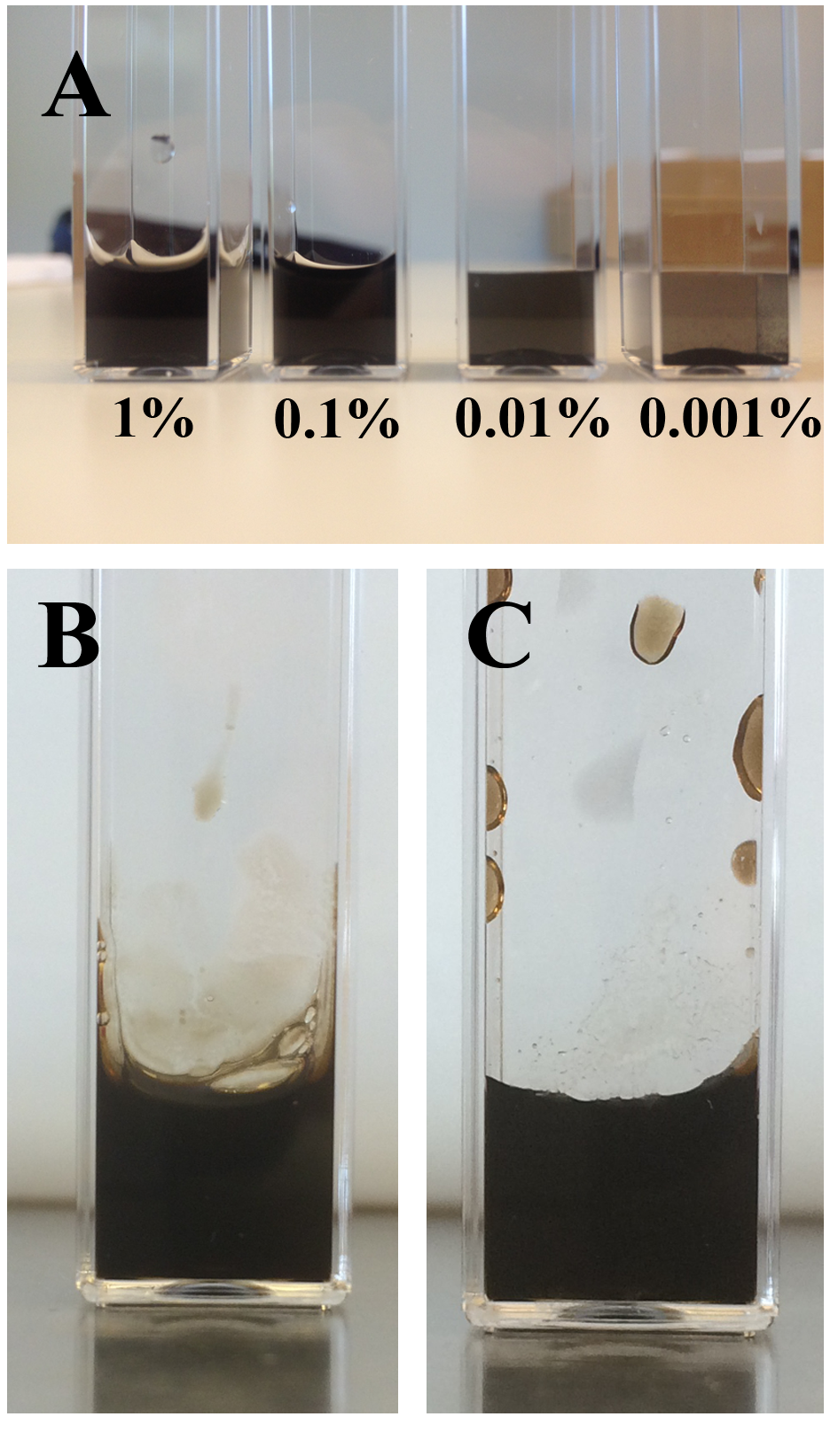

Supplement: S5 Fig — (A) rGO (3.24 mg/ml) was prepared in water added TW80 (1%, 0.1%, 0.01% or 0.001%) to visualize the effect on sedimentation. GO added 0.1% Tween80 (B) or PBS (C) that were used in this study (0.36 mg/ml). All suspensions were sonicated for 16 minutes, as described. In general, photos were captured within 30 minutes after sonication to reflect the time used to conduct the intratracheal instillation in mice. (TIF) [file pone.0178355.s005.tif]

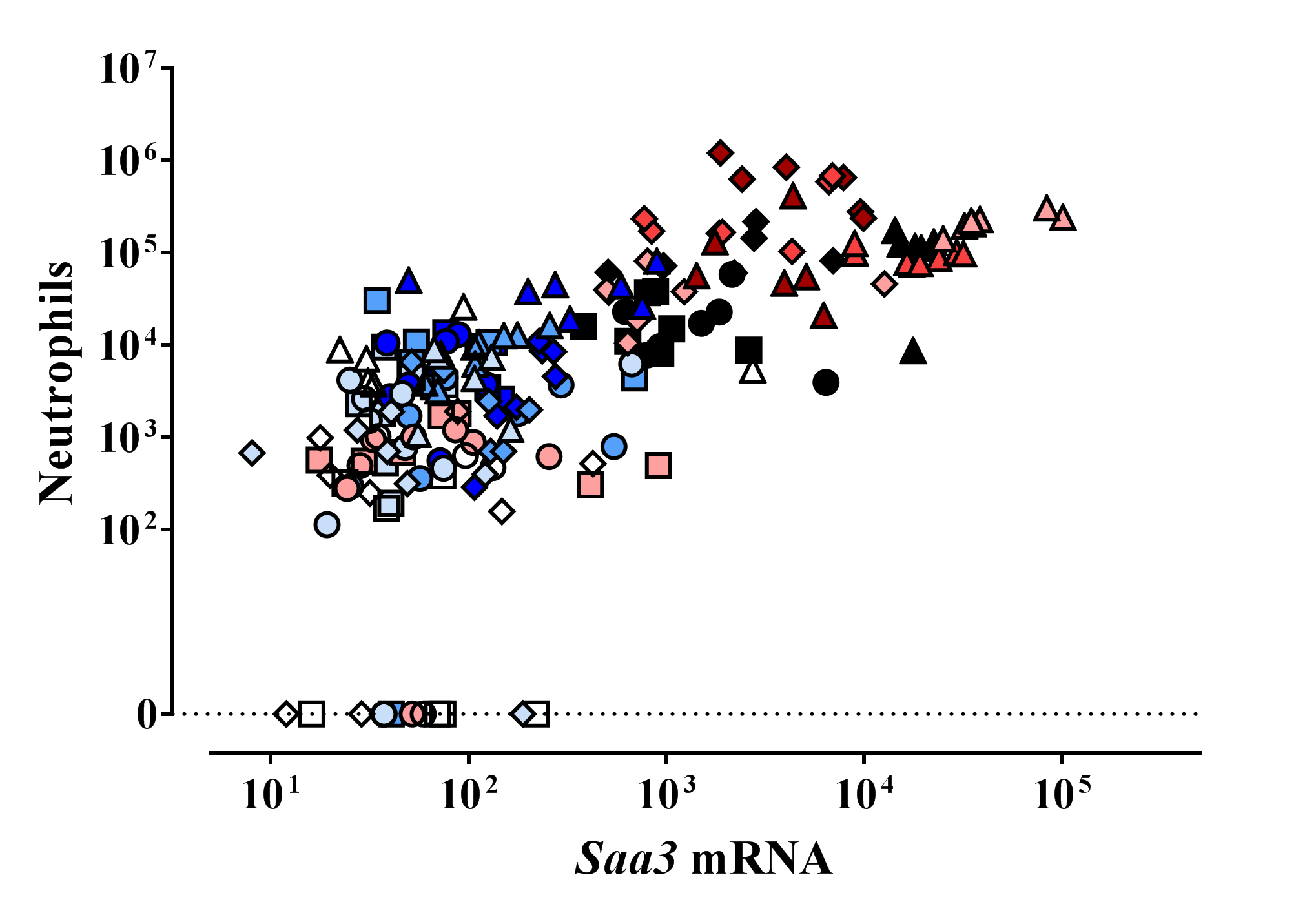

Supplement: S6 Fig — Each dot represents an individual mouse (n = 195) exposed to either VC (white), GO (red), rGO (blue) or P90 (black). Color intensity denotes dose levels (18, 54 or 162 μg/mouse), where darker colored dots denotes higher levels. Triangles, diamonds, circles and squares denotes day 1, 3, 28 and 90, respectively. (TIF) [file pone.0178355.s006.tif]

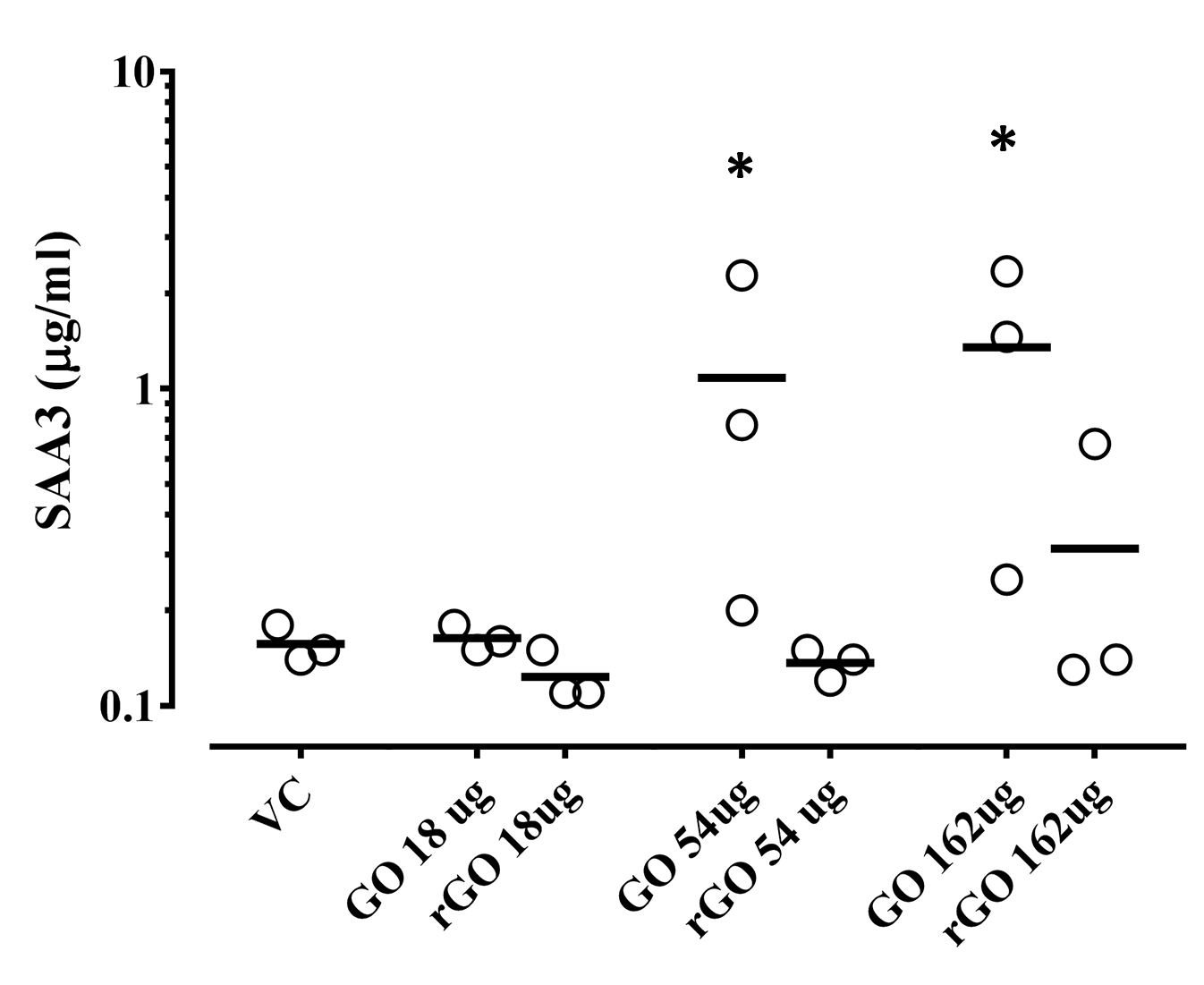

Supplement: S7 Fig — Samples in each group were pooled randomly to a final n = 3 (representing 6 samples). Black lines denote mean values.*, ** and ***: Statistically significantly different from VC at p < 0.05, p < 0.01, p < 0.001 level, respectively. (TIF) [file pone.0178355.s007.tif]

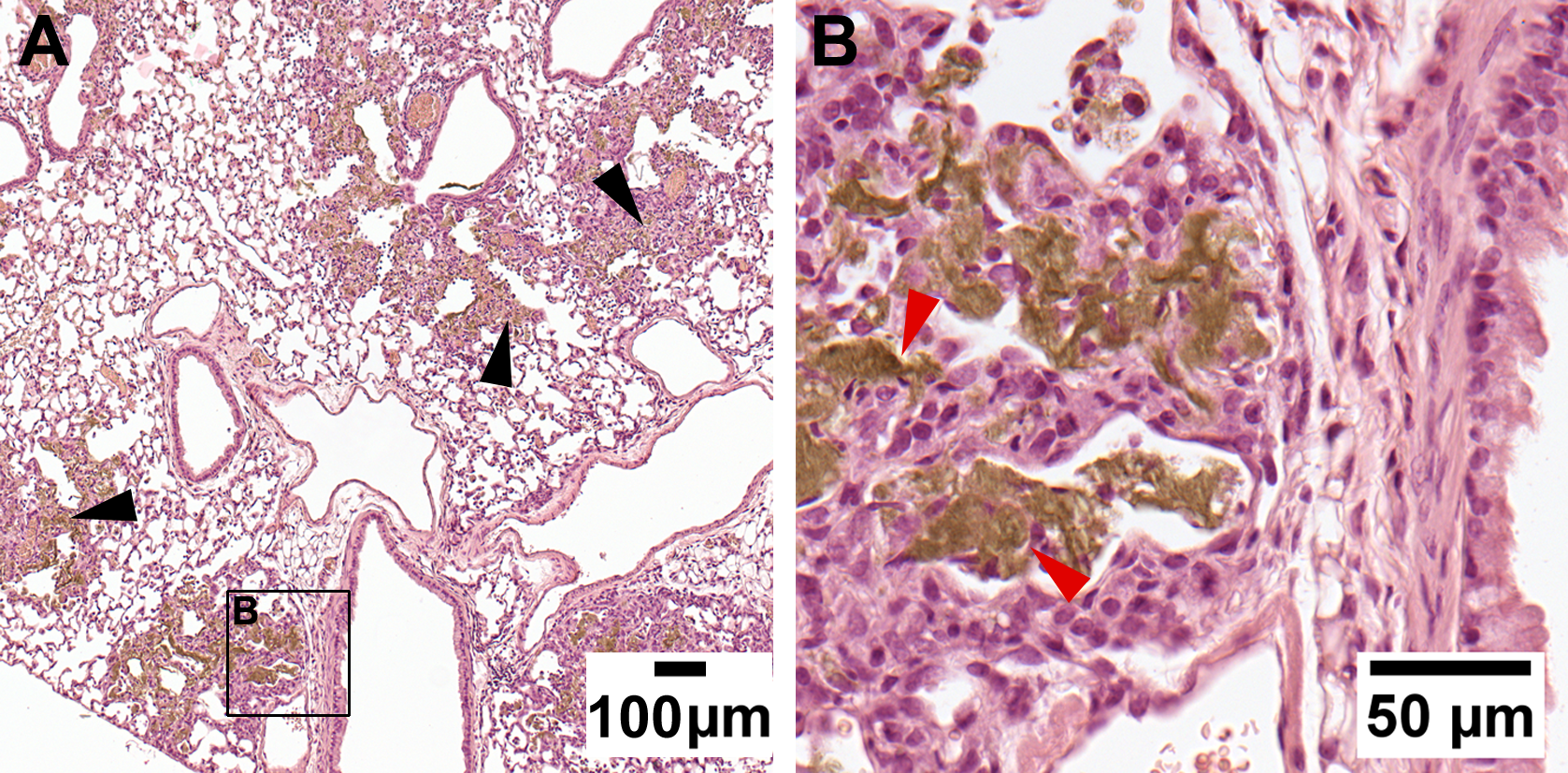

Supplement: S8 Fig — (A) Patchy appearance of acute pulmonary inflammation in areas with GO deposits (black arrows). (B) GO appeared as free light-brown granular pigments (red arrows). (TIF) [file pone.0178355.s008.tif]

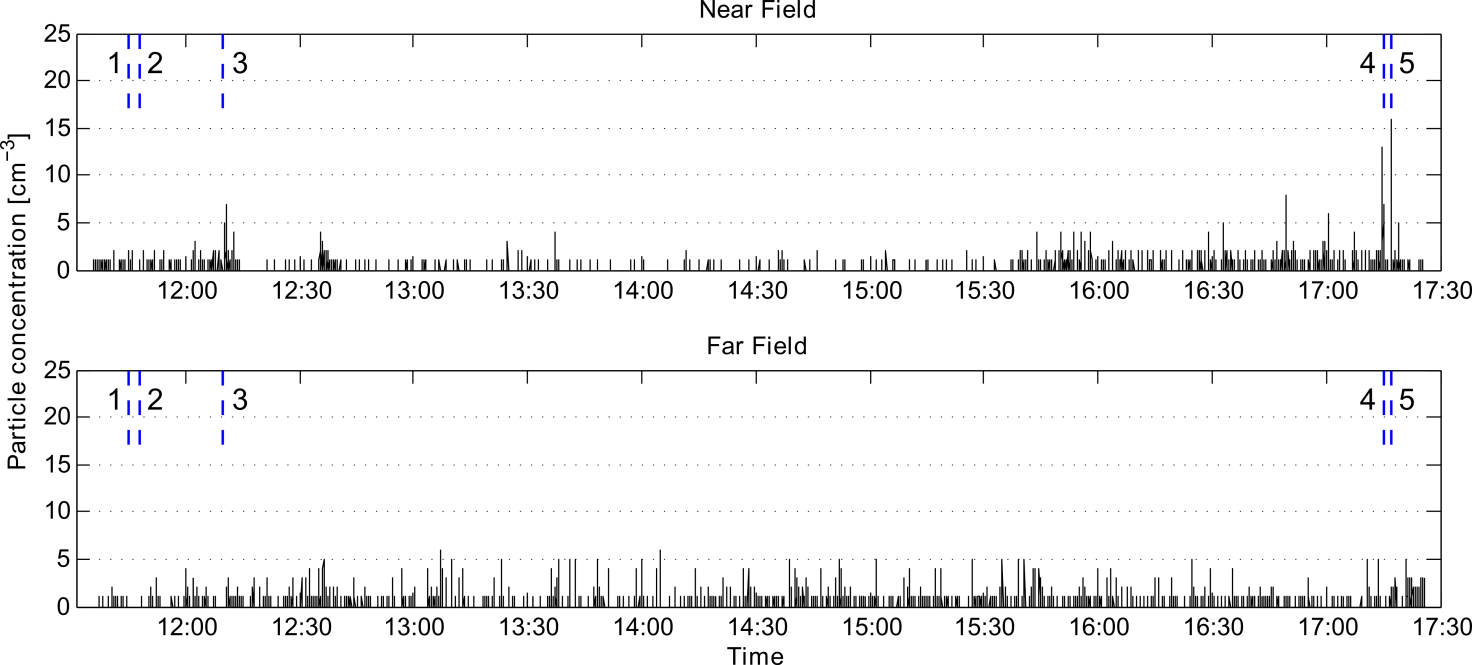

Supplement: S9 Fig — Measurements were conducted with CPC and DiSCmini in Near Field (NF) and Far Field (FF) during a work day with graphene production using chemical vapour deposition. In-graph numbers refers to time events: (1) Open reactor, (2) Close reactor, (3) Initiate growth, (4) Open reactor, (5) Dry wiping the reactor. (TIF) [file pone.0178355.s009.tif]

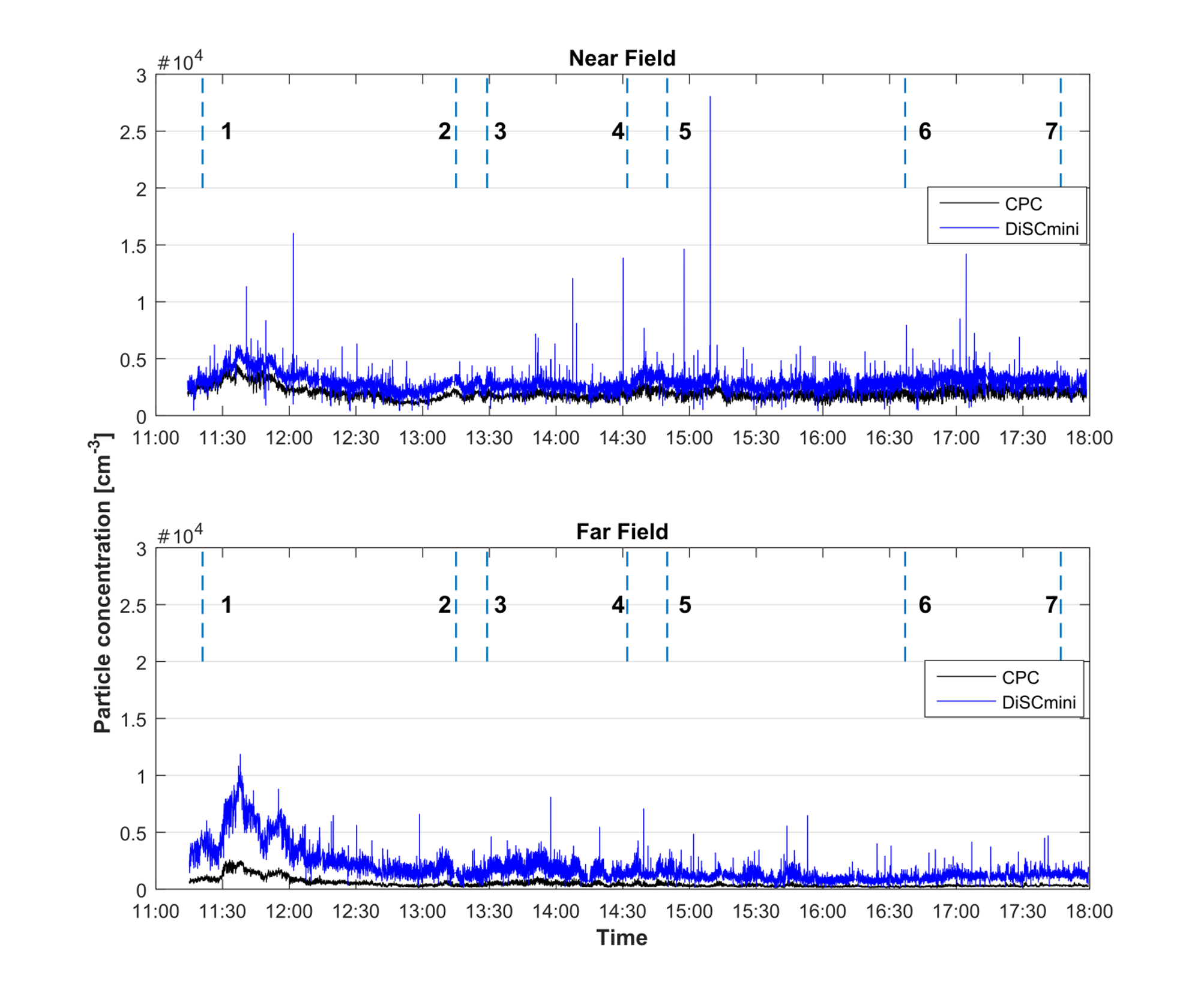

Supplement: S10 Fig — Measurements were conducted with CPC and DiSCmini in Near Field (NF) and Far Field (FF) during a work day with graphene production using chemical vapour deposition. In-graph numbers refers to time events: (1) Reactor Warm-up, (2) Open reactor, (3) Initiate growth, (4) Open nearby CNT chamber, (5) Open reactor–Wafer out, (6) Open reactor–Wafer out, (7) Open reactor–Wafer out. (TIF) [file pone.0178355.s010.tif]
