# Supplementary material for: Differences in inflammation and acute phase response but similar genotoxicity in mice following pulmonary exposure to graphene oxide and reduced graphene oxide
Source: PLoS One. 2017 Jun 1;12(6):e0178355. doi: 10.1371/journal.pone.0178355 (PMC5453440; doi:10.1371/journal.pone.0178355)
Supplement: S1 Table — Continuous bodyweight (g) measurements of mice exposed to VC, GO, rGO or P90. (DOCX) [file pone.0178355.s011.docx]

**S1 Table. Bodyweight**. Continuous bodyweight (g) measurements of mice exposed to VC, GO, rGO or P90.

|  | **Dose** | **Instillation** | **Day1** | **Day3** | **Day 28** | **Day 90** |
| --- | --- | --- | --- | --- | --- | --- |
| **VC** | **0** | 19.6 ± 0.4 (32) | 19.2 ± 0.5 (16) | 19.4 ± 0.4 (8) | 21.8 ± 4.5 (12) | 24.3 ± 0.1 (6) |
| **GO** | **18** | 20 ± 0.4 (28) | 20.0 ± 0.4 (7) | 20.2 ± 0.3 (14) | 22 ± 5.3 (14) | 24.3 ± 0.6 (7) |
|  | **54** | 20 ± 0.3 (28) | 18.0 ± 0.3 (7) | 17.2 ± 0.6 (21) | - | - |
|  | **162** | 19.7 ± 0.4 (28) | 17.4 ± 0.3 (7) | 15.2 ± 0.4 (21) | - | - |
| **rGO** | **18** | 19.3 ± 0.5 (28) | 19.4 ± 0.5 (7) | 19.1 ± 0.7 (7) | 21.2 ± 2.6 (7) | 23.8 ± 0.4 (7) |
|  | **54** | 19.5 ± 0.4 (28) | 19.6 ± 0.5 (7) | 19.8 ± 0.3 (7) | 21 ± 2.6 (7) | 23.2 ± 0.4 (7) |
|  | **162** | 19.5 ± 0.4 (28) | 19.2 ± 0.5 (7) | 19.5 ± 0.4 (7) | 21.8 ± 5.3 (14) | 23.3 ± 0.7 (7) |
| **P90** | **162** | 19.3 ± 0.4 (28) | 19.3 ± 0.4 (7) | 19.4 ± 0.4 (7) | 22.6 ± 2.6 (7) | 24.6 ± 0.6 (7) |

Bodyweight as mean ± SEM (*n*).
