# Supplementary material for: Differences in inflammation and acute phase response but similar genotoxicity in mice following pulmonary exposure to graphene oxide and reduced graphene oxide
Source: PLoS One. 2017 Jun 1;12(6):e0178355. doi: 10.1371/journal.pone.0178355 (PMC5453440; doi:10.1371/journal.pone.0178355)
Supplement: S2 Table — Differential BAL cell count (x103) from mice at 3 post exposure to 0.1% TW80, 18 μg/mouse GO in 0.1% TW80, 0.1% PBS or 18 μg/mouse GO in PBS. (DOCX) [file pone.0178355.s012.docx]

**S2 Table. BAL cell counts**. Differential BAL cell count (x10^3^) from mice at 3 post exposure to 0.1% TW80, 18 μg/mouse GO in 0.1% TW80, 0.1% PBS or 18 μg/mouse GO in PBS.

|  | **Dose** | **Neutrophils^a^** | **Macrophages^a^** | **Eosinophils** | **Lymphocytes** | **Total cells^a^** |
| --- | --- | --- | --- | --- | --- | --- |
| **VC_TW80_** | **0** | 2.5 ± 1.3 | 349.3 ± 34.4 | 6.1 ± 1.4 | 6.1 ± 2.6 | 364.1 ± 36.3 |
| **GO_TW80_** | **18** | 162.9 ± 37.3** | 547.7 ± 33.4* | 236.3 ± 88.8 | 28.8 ± 4.9 | 975.7 ± 131.3*** |
| **VC_PBS_** | **0** | 23.9 ± 19.7 | 489.4 ± 73.2 | 6.9 ± 2.7 | 9.3 ± 1.5 | 431.6 ± 67.1 |
| **GO_PBS_** | **18** | 260.5 ± 27.5* | 764.1 ± 61.1* | 202.4 ± 81.3 | 44.8 ± 14.7 | 1271.8 ± 154.8*** |

Mean ± SEM, *n*=6

^a^ Statistical analysis was performed on neutrophils, macrophages and total cells.

*, ** and ***: statistically significantly different from VC at *p* < 0.05, *p* < 0.01, *p* < 0.001 level, respectively.
